# Bile acids as therapeutic agents

**DOI:** 10.3389/fphar.2025.1732854

**Published:** 2026-01-12

**Authors:** Brandon Vu, Ryo Kawamoto, Priscila Villalba-Davila, Xinzhong Dong, Wikrom Karnsakul

**Affiliations:** 1 Solomon H. Snyder Department of Neuroscience, Johns Hopkins University School of Medicine, Baltimore, MD, United States; 2 Johns Hopkins University School of Nursing, Baltimore, MD, United States; 3 Division of Pediatric Gastroenterology, Hepatology, and Nutrition, Department of Pediatrics, Johns Hopkins University School of Medicine, Baltimore, MD, United States

**Keywords:** bile acid therapy, bile acids, bilosomes, cholestasis, FXR, neuroprotection, TGR5, UDCA

## Abstract

Bile acids (BAs) are amphiphilic molecules traditionally recognized for their role in lipid digestion but have gained increased interest for their therapeutic potential. Among them, ursodeoxycholic acid (UDCA) is the most widely prescribed and has been FDA-approved in the treatment of primary biliary cholangitis (PBC), the most common chronic cholestatic liver disease, while also being used off-label in multiple other disorders. The therapeutic effects of BAs are linked to their capacity to modulate signaling pathways, reduce hepatocellular injury, and regulate inflammation. Their physicochemical properties, particularly hydrophobicity, influence both efficacy and toxicity, of which the mechanisms involving receptors such as farnesoid X receptor (FXR), vitamin D receptor (VDR) and Takeda G protein-coupled receptor 5 (TGR5) help to explain. Recent regulatory milestones include the FDA-approval of chenodeoxycholic acid (CDCA) in the treatment of cerebrotendinous xanthomatosis (CTX) and ongoing clinical trials such as that of norucholic acid (NCA) in the treatment of primary sclerosing cholangitis (PSC). Expanding research is redefining the BA therapeutic landscape, with applications spanning cholestatic, metabolic, and neurodegenerative diseases. This review will explore established and emerging BA-based monotherapies, combination regimens, and novel BA-driven drug delivery systems.

## Introduction

Bile acids (BAs), also referred to as bile salts (BSs) in their conjugated form, are the major organic constituents of bile in mammals and other vertebrates and play essential roles in digestion, endocrine signaling, immune homeostasis, gut health and metabolic functioning (Houten et al., 2006; Hofmann and Hagey, 2008; Chiang, 2013; Faustino et al., 2016). Beyond their well-established role in lipid absorption, accumulating evidence shows that BAs act as bioactive, endogenous ligands that regulate gene expression and cellular function by activating prominent nuclear receptors such as farnesoid X receptor (FXR) and vitamin D receptor (VDR) and by mediating signaling through membrane G protein-coupled receptors (GPCRs) like Takeda G protein-coupled receptor 5 (TGR5) (Chiang, 2013; Li and Chiang, 2014; Fuchs et al., 2025). Disruption in BA synthesis, storage and circulation can cause a wide spectrum of pathophysiological consequences if left untreated. Consequently, there is growing interest in harnessing BAs as therapeutic agents for a diverse range of disorders. This review will examine the mechanisms of BA synthesis, cytotoxicity, and therapeutic application in several cholestatic and non-cholestatic diseases, as well as explore emerging evidence for their use in neurodegenerative disorders and their efficacy as a drug delivery system.

### BA synthesis and circulation

**FIGURE 1:**
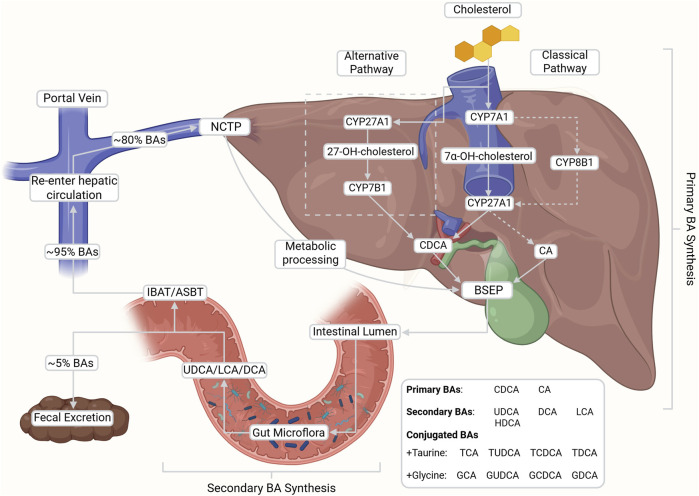
*De novo* synthesis, enterohepatic circulation, and microbial transformation of bile acids (BAs). Primary BAs, cholic acid (CA) and chenodeoxycholic acid (CDCA), are synthesized from cholesterol via the classical (CYP7A1-initiated) and alternative (CYP27A1-initiated) pathways in the liver. Upon conjugation with glycine or taurine, BAs are secreted into the intestine where they facilitate lipid digestion and are subsequently reabsorbed into portal circulation (∼95%) via the ileal bile acid transporter (IBAT/ASBT) and into the hepatocytes via sodium taurocholate co-transporting polypeptide (NTCP). After metabolic processing, BAs are reintroduced into the bile via bile salt export pumps (BSEP) at the bile canaliculi. A small fraction escapes reabsorption and is modified by gut microbiota into secondary BAs (e.g., DCA, LCA, UDCA), contributing to the recycling and regulatory feedback of the enterohepatic system. This figure was created with BioRender.com.

In humans, the multi-enzymatic process of *de novo* synthesis of BAs occurs in one of two main pathways and is regulated via negative feedback by BA-activated FXR (Shulpekova et al., 2022; Fuchs et al., 2025). BA synthesis represents a major route of cholesterol catabolism (Schwarz, 2004; Chiang, 2013; Li and Chiang, 2014; di Gregorio et al., 2021). The “classical” or neutral pathway, responsible for ∼90% of primary BA production, is initiated and rate-limited in the hepatocytes by cholesterol 7α-hydroxylase (CYP7A1) (Faustino et al., 2016; Chiang and Ferrell, 2020; di Gregorio et al., 2021; Collins et al., 2023). Hydroxylation at the C7 position yields the intermediate, 7α-hydroxycholesterol, which is then acted upon by sterol 27-hydroxylase (CYP27A1) to generate chenodeoxycholic acid (CDCA), or alternatively by 12α-hydroxylase (CYP8B1) before the CYP27A1 step to produce cholic acid (CA) (Figure 1) (Chiang, 2009; di Gregorio et al., 2021).

The “alternative” or acidic pathway, which accounts for ∼10% of primary BA synthesis, is initiated by mitochondrial CYP27A1 in both hepatic and extrahepatic tissues (Schwarz, 2004; di Gregorio et al., 2021). This hydroxylates cholesterol at the C27 position to produce the oxysterol intermediate 27-hydroxycholesterol, which is subsequently modified by oxysterol 7α-hydroxylase (CYP7B1) to generate CDCA (Schwarz, 2004; Chiang, 2009; di Gregorio et al., 2021; Shulpekova et al., 2022; Collins et al., 2023). Both pathways yield amphiphilic molecules with distinct hydrophilic and hydrophobic domains (Hofmann, 1999).

The major primary BAs (CA and CDCA) are predominantly conjugated with glycine and taurine as BSs (Chiang, 2013; Faustino et al., 2016). Conjugation is capable of lowering pKa, increasing hydrophilicity, reducing cytotoxicity, and enhancing solubility under physiological conditions (Faustino et al., 2016; Shulpekova et al., 2022). These properties improve lipid emulsification and facilitate mixed micelle formation in the small intestine (di Gregorio et al., 2021; Collins et al., 2023). Conjugated BAs are stored in the gallbladder during fasting and are released into the duodenum in response to meals (Schwarz, 2004; Chiang, 2009; Faustino et al., 2016). In addition to their digestive role, BAs have also been implicated in stimulating postprandial insulin secretion, thereby contributing to glucose metabolism (Li et al., 2012; Chiang and Ferrell, 2020). In the intestine, gut microbiota deconjugate and transform primary BAs into secondary BAs such as deoxycholic acid (DCA), lithocholic acid (LCA), hyodeoxycholic acid (HDCA) and ursodeoxycholic acid (UDCA) (Faustino et al., 2016; Shulpekova et al., 2022). More recently, gut microbiota are reported to deconjugate and transform primary BAs with amino acids besides glycine and taurine, producing microbial amino-acid-conjugated bile acids (MABAs), although their physiological significance remains to be elucidated (Quinn et al., 2020; Lin et al., 2025).

Approximately 95% of BAs are reabsorbed, with the majority of conjugated BA reuptake occurring in the ileum through the ileal bile acid transporter (IBAT), also known as the apical sodium-dependent bile acid transporter (ASBT), located on the brush border membrane of enterocytes (Chiang, 2009; 2013; Shulpekova et al., 2022). A smaller fraction of protonated, unconjugated BAs are largely absorbed passively along the colon with only about 5% escaping reabsorption for subsequent excretion in feces (Chiang, 2009; 2013; Shulpekova et al., 2022). Around 80% of these primary and secondary BAs then return to the liver from portal blood circulation via sodium taurocholate co-transporting polypeptide (NTCP) activity along the basolateral membrane of hepatocytes (Tan et al., 2024). This crucial reuptake maintains BA levels in systemic circulation and allows for the recycling and reuse of BAs in enterohepatic circulation (Hofmann and Hagey, 2008; Tan et al., 2024). From here, BAs are resecreted into the bile canaliculi via bile salt export pumps (BSEP) to reintegrate into bile (Kubitz et al., 2012). Notably, dysfunction in the regulation or expression of the BA transport proteins IBAT/ASBT, NTCP and or BSEP have been linked with numerous pathologies that will be discussed later in this review. BAs lost to excretion are replenished by *de novo* synthesis to maintain consistent levels within the body (Chiang, 2009, Ching, 2013).

Upon reentry to circulation in humans, both the conjugated and free forms of BA bind to FXR to regulate further production via one of two pathways (di Gregorio, Cautela and Galantini, 2021; Shulpekova et al., 2022). In the first, BA-bound FXR in ileal enterocytes induces the production of the endocrine peptide fibroblast growth factor 19 (FGF19), which then activates hepatic FGF receptor 4 (FGFR4), resulting in the suppression of CYP7A1 expression and inhibition of the classical BA synthesis pathway (Chiang, 2009; Shulpekova et al., 2022). In the second pathway, BA-bound hepatic FXR forms a complex with retinoid X receptor to produce a small heterodimer partner, leading to suppression of CYP7A1 and CYP8B1 (di Gregorio et al., 2021; Shulpekova et al., 2022). In both pathways, FXR acts as a critical inhibitory receptor to regulate the over or underproduction of BAs (Schwarz, 2004; Chiang, 2009; di Gregorio et al., 2021).

Circulating BAs have also recently been implicated as important signaling metabolites and mediators of neurological disease in both the brain and blood brain barrier (BBB) (Ferrell and Chiang, 2021). The crosstalk between the metabolic functions of the liver, microbial modification and conjugation of BAs in the gut and cellular signaling of BAs within the brain constitute what is known as the gut-liver-brain axis (GLBA) (Ferrell and Chiang, 2021; Sun et al., 2024). Communication between these three systems impact numerous physiological processes including host immunity modulation, metabolism and neurodevelopment (Sun et al., 2024). Blood BAs are also able to cross the BBB to act directly or indirectly with receptors in the brain, though disruption to serum BA levels due to hepatointestinal pathology can influence systemic inflammatory responses, BBB permeability, and neuronal synaptic functioning (Yeo et al., 2023; Sun et al., 2024). Notably, increased serum BA levels have also been found to modulate the permeability of both the BBB through the compromise of tight junctions and gut vascular barrier via the FXR receptor (Quinn et al., 2014; Sun et al., 2024). This suggests interesting implications for the involvement of BA in neurodegenerative disease and its potential role as a therapeutic agent. Current knowledge regarding the mechanisms by which BAs affect the BBB are under investigation.

## BAs as pathogenic agents

### Pathogenesis of BA-associated diseases and symptoms

Dysfunction in the synthesis, storage or circulation of BAs can result in a range of physiological disturbances. Disorders arising from these defects in enterohepatic circulation are generally referred to as cholestatic diseases or cholestasis (Hasegawa et al., 2021; Beuers et al., 2023). This is defined as the impairment of BA flow resulting in a toxic accumulation of BAs and BA metabolites in the liver and systemic circulation (Li and Apte, 2015). Such impairments may be driven by genetic, hormonal, hepatobiliary, metabolic, exogenous, autoimmune or alloimmune factors (Li and Apte, 2015; Sarcognato et al., 2021).

Although the underlying pathophysiology varies, cholestasis can lead to inflammation, fibrosis, cirrhosis, hepatocyte dysfunction, hepatocellular carcinoma, cholangiocarcinoma, and ultimately end-stage liver disease (ESLD) requiring transplantation (Li and Apte, 2015; Sarcognato et al., 2021). Mechanical or structural obstructions to BA flow may further complicate disease progression (Cariello et al., 2022).

In response to rising intrahepatic concentrations of hydrophobic and hepatotoxic BAs, hepatocytes upregulate BA sulfation as a major adaptive detoxification pathway (Alnouti, 2009). Sulfotransferase-mediated conjugation increases BA solubility and reduces membrane-disruptive properties, promoting basolateral efflux and renal elimination (Alnouti, 2009). As a result, sulfated BAs function as a promising marker for distinguishing types of liver injuries and identifying BA overload as well as a protective mechanism to prevent further hepatic damage (Alnouti, 2009; Masubuchi et al., 2016). Dysregulated sulfation has been linked to increased disease severity in cholestasis and contributes to altered immune signaling (Alnouti, 2009; Ito et al., 2024).

Clinically, cholestasis often presents with jaundice due to hepatic insufficiency, persistent fatigue, features of sicca complex, and most notably, pruritus which is reported in up to 80% of affected individuals, which remains one of the most burdensome symptoms and a notable adverse effect of therapeutic BA usage (Beuers et al., 2014; Hirschfield et al., 2017; Beuers et al., 2023). Given this dual unique role of BAs in both disease causation and therapy, understanding their toxic potentials is essential to maximizing benefit and minimizing harm.

### BA toxicity and hydrophobicity

While all BAs are amphiphilic, their hydrophobic-hydrophilic balance influences digestive efficacy and cytotoxic potential (Perez and Briz, 2009). The same hydrophobicity that enables effective micelle formation for lipid digestion, also facilitates its interaction with nonpolar cell membranes (Hofmann, 1999). Highly hydrophobic BAs, such as LCA, can disrupt lipid bilayers, increasing membrane permeability and promoting apoptosis (Perez and Briz, 2009). In cholestasis, progressive accumulation of hydrophobic BAs within the bile has experimentally been shown to induce hepatocyte injury, necrosis and subsequent liver damage (Hofmann, 1999; Amaral et al., 2009; Perez and Briz, 2009). This process frequently involves mitochondrial dysfunction characterized by membrane disruption, generation of reactive oxygen species (ROS) and mitochondrial permeability transition (MPT) (Perez and Briz, 2009). As a result of this, the liver relies heavily on BA sulfation which markedly increases hydrophilicity, lowers detergent activity, and enhances solubility in biological fluids to reduce BA overload (Alnouti, 2009).

ROS production activates nuclear factor-κB (NF-κB) signaling via IκB kinase (IKK) activation, driven by upstream mediators such as tumor necrosis factor α (TNF-α) (Lingappan, 2018). Activated NF-κB translocates to the nucleus, promoting transcription of pro-inflammatory, pro-oxidant and anti-apoptotic genes (e.g., Bcl-2 and Bcl-xL) and upregulating inflammatory cytokines such as IL-1β, IL-6 and TNF-α (Catz and Johnson, 2001; Liu et al., 2017; Lingappan, 2018). Notably, prolonged or chronic ROS production can eventually inhibit NF-κB activity, while sustained inflammation further enhances oxidative stress, creating a self-reinforcing cycle of injury (Liu et al., 2017; Forrester et al., 2018).

At pathophysiological concentrations, hydrophobic BAs can also activate transmembrane death receptors such as Fas and TNF-related apoptosis-inducing ligand receptor (TRAILR) (Wei et al., 2020). This recruits the Fas associated death domain (FADD), subsequently leading to the activation of caspases, a family of specialized proteases that execute programmed cell death (Amaral et al., 2009). Caspase-8, the main initiator of death receptor signaling, cleaves cytoplasmic Bid, which activates and induces conformational changes in the Bax and Bak proteins to relay the apoptotic signal to the mitochondria (Amaral et al., 2009; McArthur and Kile, 2018). Bax/Bak pore formation in mitochondrial membranes leads to cytochrome *c* release into the cytosol, apoptosome assembly with Apaf-1, and activation of caspase-9 and the effector caspases-3, -6, and -7, culminating ultimately in cell death (Amaral et al., 2009; Zhang M. et al., 2017; McArthur and Kile, 2018).

BA activation of TGR5 has been linked to pro-proliferative mechanisms that possess implications in both cancerous and regenerative settings (Pols et al., 2011; Guo et al., 2016). Varied findings have observed that TGR5 was overexpressed in cancers such as esophageal adenocarcinoma but suppressed the proliferation of gastric cancer cells, suggesting a potential tumorigenesis involvement (Guo et al., 2016). Elevated serum BA levels have also been found to enhance the proliferation of cholangiocarcinoma cells in animal models through a proposed TGR5-mediated mechanism (Reich et al., 2016).

The nuclear receptor VDR plays a critical role in hydrophobic BA regulation. Although best known as the mediator of the calcemic hormone 1α,25-dihydroxyvitamin D_3_ (1,25(OH)_2_D_3_), VDR is also a high-affinity receptor for the secondary BA, LCA (Makishima et al., 2002; Pavek et al., 2010; Cheng et al., 2014). Activation of VDR by 1,25(OH)_2_D_3_ or LCA induces the expression of *in vivo* human cytochrome P450 3A4 (CYP3A4), a critical enzyme involved in the biotransformation of 50% of drugs as well as Vitamin D_3_ (Pavek et al., 2010; Kasarla et al., 2022). Although the pregnane X receptor (PXR) is the dominant regulator of CYP3A4 induction in the liver, VDR also transactivates CYP3A4 in the intestine and is broadly expressed across many tissues (Cheng et al., 2014). CYP3A4 hydroxylates LCA, reducing its hydrophobicity while also suppressing intestinal BA transporter expression, inhibiting CYP7A1 to limit further BA synthesis and reducing inflammation through the regulation of the NF-κB pathway (Han and Chiang, 2009; Cheng et al., 2014; Dong et al., 2020). In multiple studies, VDR-deficient mice show markedly worsened LCA-induced hepatotoxicity, altered body and colonic morphology, increased hepatic inflammation and heightened susceptibility to inflammatory bowel disease (IBD) compared to controls, underscoring VDR’s importance in safeguarding against cholestatic injury (Cheng et al., 2014; Dong et al., 2020). Notably, high levels of LCA that activate VDR may limit its practical therapeutic capacity. However, recent studies have explored the potential of LCA derivatives such as LCA acetate which display greater potency and improved safety profiles compared to LCA itself (Adachi et al., 2005; Sasaki et al., 2021).

**FIGURE 2:**
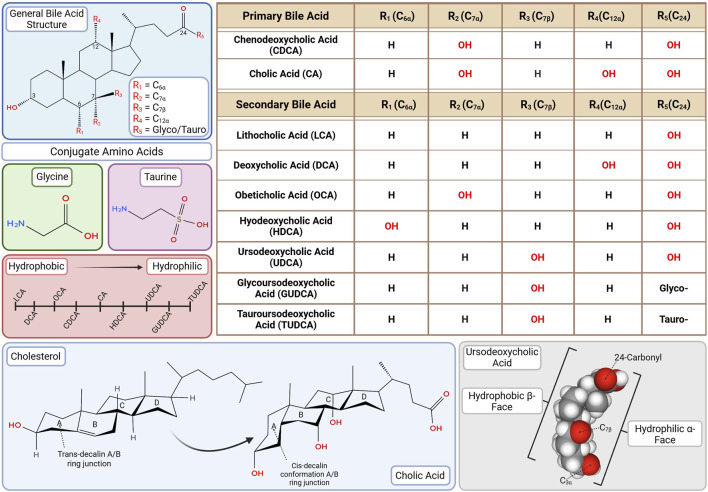
Bile acid (BA) structure and hydrophilicity. The number, degree, position and stereochemistry of hydroxyl groups on the steroid nucleus (especially at C3, C6, C7, and C12) determine hydrophilic-hydrophobic balance, influencing toxicity and receptor binding. Glycine or taurine conjugation further moderates solubility, enterohepatic circulation, kinetics and receptor binding affinity. This diversity underpins the rationale for selective therapeutic application ranging from FXR agonist-based interventions with OCA to neuroprotective strategies using GUDCA and TUDCA. This figure was created in BioRender.com. The chemical structures were created in app.molview.com.

Hydrophobicity is dictated by the number, degree and position of hydroxylation on the cholesterol backbone, orientation of hydroxyl groups, and the nature of glycine or taurine conjugation (Thomas et al., 2008; Perez and Briz, 2009). Hydrophilic BAs, such as UDCA, glycoursodeoxycholic acid (GUDCA) and tauroursodeoxycholic acid (TUDCA), are favored therapeutically for their lower toxicity and broad clinical utility (Paumgartner and Beuers, 2002; Pusl and Beuers, 2006; Perez and Briz, 2009; Khalaf et al., 2022). In contrast, moderately hydrophobic BAs like CDCA and its semi-synthetic derivative obeticholic acid (OCA) are potent FXR agonists and used to modulate BA synthesis in selected cholestatic conditions (Liu et al., 2014; Zhang Y. et al., 2017). With EC_50_ values of ∼10 μM for CDCA and ∼100 nM for OCA, the latter’s greater potency has expanded its use despite higher toxicity risk at elevated doses though, notably, OCA has been banned for therapeutic usage in Europe (Zhang Y. et al., 2017; Yang et al., 2024). Hydrophobicity hierarchy (most to least) is: LCA > DCA > OCA > CDCA > CA > HDCA > UDCA > GUDCA > TUDCA (Figure 2) (Heuman, 1989; Perez and Briz, 2009).

Notably, usage of more hydrophobic BAs, such as OCA, is predictably reported to cause BA-induced pruritus (Nevens et al., 2016), making it one of the most frequent and dose-limiting adverse effects of BAs that significantly impair patient quality of life. Although its molecular mechanism remains incompletely understood, our group and others have identified Mas-related G protein-coupled receptor X4 (MRGPRX4), a human GPCR specifically expressed in peripheral itch sensing neurons, as a receptor for BA and bilirubin that potentially mediate BA-induced pruritus (Meixiong et al., 2019; Meixiong, 2025; Yu et al., 2019). However, other studies have questioned this model, reporting that BSs and bilirubin activate MRGPRX4 or other known pruriceptors only at supraphysiological concentrations, and that albumin at physiological levels abolishes such activation, suggesting these metabolites may not directly drive cholestatic itch (Wolters et al., 2025).

## BAs as therapeutic agents

### Therapeutic mechanisms of BAs

Given the harmful effects of cholestasis, certain BAs demonstrate their therapeutic benefits via multiple mechanisms to address a variety of conditions. UDCA in particular is proposed to act through three primary therapeutic mechanisms (Paumgartner and Beuers, 2002).

First, it protects cholangiocytes from BA-induced cytotoxicity by lowering the concentration of highly hydrophobic BAs that can accumulate and cause cellular necrosis in cholangiocytes and hepatocytes (Paumgartner and Beuers, 2002; Perez and Briz, 2009). This reduction also limits the activation of both ligand-dependent and ligand-independent apoptotic death receptors by cytotoxic BA buildup and oxidative stress (Paumgartner and Beuers, 2002; Amaral et al., 2009; Perez and Briz, 2009).

Second, immunomodulative properties activate cell survival signaling pathways that both attenuate apoptosis and prevent mitochondrial dysfunction (Amaral et al., 2009). While unbound FXR exhibits anti-apoptotic effects through the inhibition of caspase-8, BA–activated FXR has also been shown to protect against apoptosis induced by BA overload and deoxysphingolipids, while simultaneously modulating the NF-κB-mediated inflammatory response (Fleishman and Kumar, 2024). This occurs through the promotion of Cytochrome P450 Family 4 Subfamily F (CYP4F)-mediated metabolism of the pro-apoptotic deoxysphingolipids to prevent apoptosis (Fleishman and Kumar, 2024). BA overload is inhibited through the suppression of CYP7A1 and CYP8B1 expression, thereby helping to alleviate cholestasis through the reduction of BA synthesis (Fleishman and Kumar, 2024). BA–activated FXR induces the nuclear receptor small heterodimer partner (SHP), which represses the transcription of these key BA synthetic enzymes, thereby reducing BA accumulation and mitigating apoptosis (Fleishman and Kumar, 2024).

Third, enhanced bile secretion in the hepatocytes and bile duct epithelial cells improve enterohepatic circulation to limit cholestasis (Paumgartner and Beuers, 2002; Amaral et al., 2009; Perez and Briz, 2009).

**FIGURE 3:**
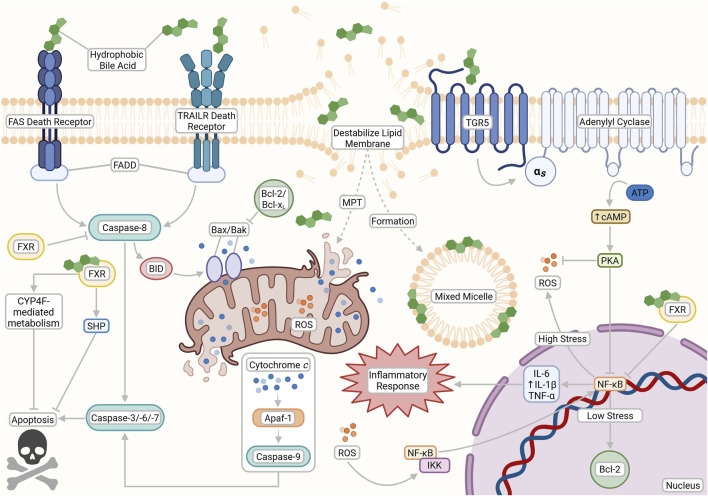
The opposing cytotoxic and cytoprotective effects of bile acids (BAs) based on their physicochemical properties and receptor interactions. Hydrophobic BAs can initiate apoptosis through both nonspecific interactions and death receptor pathways (FAS and TRAILR), leading to caspase activation, lipid membrane destabilization, mixed micelle formation, mitochondrial permeability transition (MPT), ROS production, and downstream inflammation. In contrast, cytoprotective pathways are activated by hydrophilic BAs and TGR5 signaling, which stimulates cAMP production via adenylyl cyclase to support anti-apoptotic responses and reduce oxidative stress. FXR signaling also plays a dual role by modulating BA metabolism and inhibiting apoptosis through transcriptional repression of pro-death genes. This figure was created with BioRender.com.

Recent studies have also elucidated the role of BA-activated TGR5 in NF-κB inflammatory suppression in the liver and stomach. Ligand-bound TGR5 induces a structural shift that activates its coupled Gαs protein and promotes the conversion of ATP to cAMP by adenylate cyclase, resulting in elevated intracellular cAMP levels (Zangerolamo et al., 2025). The increase in cAMP activates protein kinase A (PKA), which plays a key role in suppressing NF-κB signaling by disrupting its activation and limiting inflammatory gene expression (Zangerolamo et al., 2025). TGR5 activation has also been seen to suppress the production of cytokines in both Kupffer and THP-1 cells (Guo et al., 2016). TGR5 can be activated by both conjugated and unconjugated BAs. However, the most potent activators are, ironically, the hydrophobic LCA and DCA, with EC_50_ values of 0.53 and 1.25 μM respectively, in stark contrast to the significantly weaker activation by UDCA, which has an EC_50_ of 36.4 μM (Zangerolamo et al., 2025). Notably, evidence also suggests that BA-activated TGR5 may enhance caspase-8 activation, leading to increased apoptotic signaling (Yang et al., 2007). More research is needed to clarify the therapeutic role of BA-activated TGR5 (Figure 3).

BAs have been increasingly implicated in the regulation of glycemic control and overall lipid and glucose metabolism (Zarrinpar and Loomba, 2012; Guo et al., 2016; Cadena Sandoval and Haeusler, 2025). BA activation of TGR5 has been shown to enhance the release of gut-derived incretins such as GLP-1, which stimulate insulin release and suppress glucagon production (Guo et al., 2016; Cadena Sandoval and Haeusler, 2025). Some studies suggest that hydrophilic BAs may act directly on pancreatic β-cells, exerting cytoprotective effects and promoting insulin secretion via TGR5-PKA signaling or through FXR-mediated induction of the TRPA1 ion channel or the FOXA2 transcription factor (Lee et al., 2010; Düfer et al., 2012). However, *in vivo* mouse models indicate that the predominant metabolic benefits of BAs are likely mediated indirectly through gut hormone release rather than direct β-cell stimulation (Kuhre et al., 2018). Further supporting their metabolic relevance, reduced circulating levels of 12α-hydroxylated BAs, including DCA, have been associated with improved insulin sensitivity in humans (Cadena Sandoval and Haeusler, 2025). TUDCA, an established inhibitor of endoplasmic reticular stress, is also currently being investigated for its role in vascular dysfunction, mitochondrial stabilization, and anti-apoptotic activity (Wu et al., 2024b).

These cytoprotective and metabolic regulatory properties have become the focus of recent research in the potential use of BAs in both metabolic and neurodegenerative diseases, which will be discussed later in this review. Lastly, therapeutic BAs have been suggested to stimulate the liver’s bile secretion by activating signaling pathways that help move transporter proteins such as BSEP and MRP2 to the cell membrane, allowing them to pump bile components more effectively (Paumgartner and Beuers, 2002). Indeed, BAs possess numerous mechanisms by which they are able to potentially treat cholestatic and non-cholestatic conditions. Therefore, this review will primarily focus on the potential therapeutic roles of BAs and their metabolites across a range of reported pathologies, examining specific treatment applications in relation to their corresponding conditions (Table 1).

**TABLE 1:** Therapeutic applications, safety profile, dosage ranges, and regulatory status of selected bile acids (BAs) and their derivatives (Figure 4).

Approval status	Bile acid/metabolite	Therapeutic use	Adverse effects	Typical dosage range	References
FDA approved agents	Deoxycholic acid (DCA)	FDA: Cosmetic submental fat reduction	Injection site reactions, swelling, pain, hypertension, erythema, pruritus	Adult: 0.2 mL subQ injection (50 injections max)	Šarenac and Mikov (2018), Liu, Li and Alster (2021)
	Obeticholic acid (OCA)	FDA: PBC (second-line) (accelerated approval) (Investigational) potential usage for PSC and NASH/MASH	Pruritus, fatigue, increased LDL, hepatotoxicity (dose-dependent)	Adult: 5–10 mg/day	Fiorucci and Distrutti (2019b), Beuers et al. (2025)
	Chenodeoxycholic acid (CDCA)	FDA: Cholestanol storage disease/CTX, gallstone dissolution (rare, historical)	Diarrhea, reversible ↑ aminotransferases, GI upset, hepatotoxicity, and hypercholesterolemia	Adult: 13–16 mg/kg/day (gallstone), 250 mg TID (CTX) Ped: 5–15 mg/kg/day in children (CTX)	Fiorucci and Distrutti (2019a), Zaccai et al. (2024)
	Cholic acid (CA)	FDA: Bile acid synthesis disorders, peroxisomal disorders (e.g., Zellweger spectrum) Off-label: CTX (if intolerant to CDCA) Familial hypertriglyceridemia, Persistent, worsening liver function or cholestasis	Diarrhea, abnormal LFTs, pruritus, rare hepatotoxicity	Adult/Ped: 10–15 mg/kg/day once or BID	Šarenac and Mikov (2018), Beuers et al. (2025)
	Ursodeoxycholic acid (UDCA)	FDA: Primary biliary cholangitis (PBC), gallstone dissolution Off-label: PSC, ICP, PFIC, BRIC, ALGS, biliary atresia, biliary cirrhosis (cystic fibrosis), cholestasis of parenteral nutrition, cholestatic jaundice syndrome, congenital dilatation of lobar intrahepatic bile duct, chronic hepatitis	Diarrhea, mild GI upset, rare hepatotoxicity	Adult: 10–12 mg/kg/day (gallstone dissolution), 13–15 mg/kg/day (PBC) Ped: 15–30 mg/kg/day (low birth weight), ≤20 mg/kg/day (cholestasis)	Cabrera, Arab and Arrese (2019), Beuers et al. (2025)
Non-FDA approved agents	Lithocholic acid (LCA)	None (not approved for any indication)	Hepatotoxicity, cholestatic injury, potential anti-inflammatory effects in colon (preclinical)	Not established	Ward et al. (2017), Lenci et al. (2023)
	Hyodeoxycholic acid (HDCA)	None (Investigational) potential usage in NAFLD/MAFLD	Not established	Not established	Kuang et al. (2023)
	Glycoursodeoxycholic acid (GUDCA)	None (Investigational) potential usage in neurodegenerative and metabolic diseases	Not established	Not established	Huang et al. (2022a), Chen et al. (2023)
	Tauroursodeoxycholic acid (TUDCA)	None (Investigational) used in some countries for cholestatic liver disease, potential usage in neurodegenerative disease	Not established; generally well tolerated in studies	Not established	Zangerolamo et al. (2021), Khalaf et al. (2022)

This table summarizes the current FDA-approved indications, common and investigational therapeutic uses, reported adverse effects, typical dosage ranges for adults and pediatric populations (where available), and regulatory approval status of naturally occurring and synthetic BAs. References correspond to key supporting literature. Dosage ranges for investigational agents are provided only when available from preclinical or early clinical studies.

**FIGURE 4:**
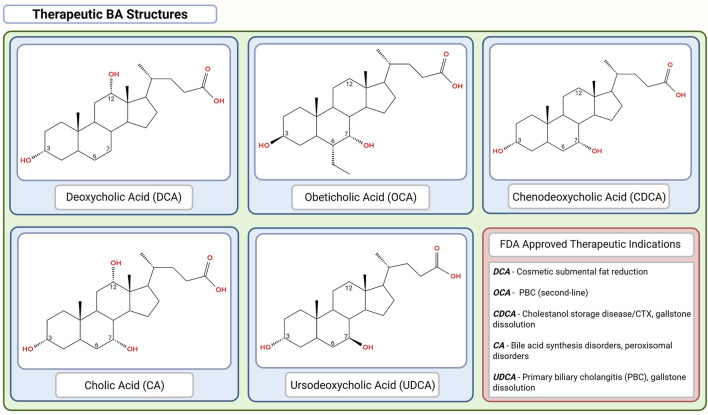
Chemical structures of the FDA-approved therapeutic bile acids (BAs). Chemical structures are organized in descending order of hydrophobicity with DCA > OCA > CDCA > CA > UDCA. This figure was created in BioRender.com. The chemical structures were created in app.molview.com.

## Therapeutic effects of BAs

### Adult cholestatic diseases

#### Primary biliary cholangitis (PBC)

PBC is a chronic autoimmune-mediated cholestatic disease characterized by loss of tolerance to the mitochondrial pyruvate dehydrogenase complex E2 (PDC-E2) which results in lymphocytic damage to intrahepatic bile duct epithelium (Hasegawa et al., 2021; Sarcognato et al., 2021; Houri and Hirschfield, 2024). It predominantly affects middle-aged women with an approximate 10:1 female-to-male ratio in reported prevalence (Sarcognato et al., 2021; Trivella et al., 2023).

The pathogenesis of PBC appears to be associated with a combination of genetic predisposition as well as specific environmental triggers such as cigarette smoking, cosmetic hair dye products, infectious agents, xenobiotics, reproductive hormone replacement and nail polish (Sarcognato et al., 2021; Levy et al., 2023; Houri and Hirschfield, 2024; Tanaka et al., 2024). The hallmark serological finding is the presence of antimitochondrial antibodies (AMA) which is present in more than 90% of cases (Levy et al., 2023).

UDCA at 13–15 mg/kg/day is considered the first-line therapy, improving transplant-free survival and delaying disease progression (Hasegawa et al., 2021; Sarcognato et al., 2021). For patients with inadequate biochemical response or intolerance to UDCA, OCA, a steroidal FXR agonist, has received accelerated FDA-approval as the second-line therapy, either in combination with UDCA or as a monotherapy (Hasegawa et al., 2021; Levy et al., 2023; Beuers et al., 2025; Fuchs et al., 2025). Dosing is generally initiated at 5 mg/day and can be increased to 10 mg/day after 3 months if liver tests remain abnormal (Hasegawa et al., 2021). OCA’s relatively high hydrophobicity makes it a more toxic alternative to UDCA and is contraindicated in patients with decompensated cirrhosis or portal hypertension due to risk of serious hepatic adverse events (Beuers et al., 2025). This, coupled with inconsistent phase IV studies have led to the European Medicines Agency to revoke marketing for the drug.

Other BA related therapies include proliferator-activated receptor (PPAR) agonists and IBAT inhibitors. Both seladelpar (PPAR-δ agonist) and elafibranor (PPAR α/δ agonist) were granted accelerated FDA-approved as well as authorization by the European Commission for the treatment of PBC; phase 3 trials have demonstrated significant improvement in biochemical response and are generally well tolerated with seladelpar also being associated with reduced pruritus (Hirschfield et al., 2024; Kowdley et al., 2024). Bezafibrate (pan-PPAR) is not FDA-approved for PBC but it is used off-label for this condition (Levy et al., 2023; Houri and Hirschfield, 2024; Beuers et al., 2025). IBAT inhibitors in PBC are intended to reduce cholestatic pruritus by blocking ileal reabsorption of BAs; these agents have not shown disease-modifying biochemical benefits comparable to FXR/PPAR agonists and are positioned for symptom control rather than disease-modifying agents (Hegade et al., 2017).

Setanaxib, a selective NADPH oxidase (NOX) 1/4 inhibitor, has shown antifibrotic potential by reducing ROS, inhibiting hepatic stellate cell activation, and myofibroblasts transformation in preclinical models (Fiorucci et al., 2024). A phase 2 trial in UDCA non-responders demonstrated reductions in ALP, liver stiffness (via elastography), and fatigue scores (Invernizzi et al., 2023). These promising findings have led to the ongoing phase 2b/3 TRANSFORM trial (NCT05014672) evaluating Setanaxib as an add-on therapy.

Foscenvivint, a selective inhibitor of the cyclic adenosine monophosphate response element-binding protein (CREB)-binding protein/β-catenin interaction, has also demonstrated antifibrotic activity in animal models of cholestatic liver disease (Kimura et al., 2022). Findings from a phase 1 trial suggested that Foscenvivint was well tolerated at the dose of 280 mg/m^2^/4 h and that it may have antifibrotic effects in advanced PBC (Kimura et al., 2022). Additional trials are required to establish efficacy and long-term safety.

Nanoparticle-based immune modulation represents an emerging therapeutic avenue in PBC. CNP-104, a biodegradable nanoparticle encapsulating the PDC-E2 antigen, is designed to induce antigen-specific immune tolerance by suppressing autoreactive T cells. A phase 2a clinical trial (NCT05104853) is currently underway to evaluate its safety and efficacy (Levy and Bowlus, 2025).

Without effective therapy, PBC can progress to cirrhosis and liver failure, with liver transplantation as the definitive treatment for advanced or decompensated disease (Hasegawa et al., 2021; Sarcognato et al., 2021).

#### Primary sclerosing cholangitis (PSC)

PSC is a rare, chronic autoimmune-mediated cholestatic liver disease characterized by fibroinflammatory injury of the biliary tree with multifocal strictures of the intrahepatic and/or extrahepatic bile ducts and periductal fibrosis leading to impaired bile flow (Karlsen et al., 2017; Dyson et al., 2018; Cazzagon et al., 2024; Manns et al., 2025). The course of the disease ranges from indolent to progressive cholestasis, often advancing to portal hypertension, cirrhosis, and liver failure (Karlsen et al., 2017; Cazzagon et al., 2024). PSC is also associated with increased risk of colorectal cancer and cholangiocarcinoma (Karlsen et al., 2017; Cazzagon et al., 2024).

While its pathogenesis remains unclear, there also exists a strong association between PSC and IBD with studies reporting that 70%–88% of PSC patients develop comorbid IBD at some point during the disease process (Cariello et al., 2022; van Munster et al., 2024). Conversely, comorbid PSC is only observed in 2%–14% of patients with IBD (van Munster et al., 2024). IBD is characterized by chronic intestinal inflammation with conditions that include diseases such as ulcerative colitis and Crohn’s Disease. Comorbidity is speculated to be associated with gut microbiome and BA profile alterations that arise due to complications related to defective BA metabolism (Thomas et al., 2022; Leibovitzh et al., 2024). PSC-IBD patients revealed lower levels of secondary conjugated BAs, including DCA and LCA, with a corresponding increase in the abundance of primary BAs compared to patients with IBD alone (Bai et al., 2024). Interestingly, these patients also tended to express milder IBD severity with one study suggesting that PSC may even attenuate the effects of IBD (Bedke et al., 2024). Even more unexpectedly, another study reported that intestinal inflammation actually improved acute cholestatic liver injury and reduced liver fibrosis in chronic colitis mouse models (Gui et al., 2023). PSC livers were also noted in one study to possess comparable levels of VDR expression to controls, suggesting another mechanism of pathology (Kempinska-Podhorodecka et al., 2017). More research is needed to fully elucidate the complicated relationship between these two conditions.

Diagnosis of PSC relies on cholestatic liver biochemistry and characteristic cholangiographic findings, typically in the absence of an identifiable cause (Karlsen et al., 2017; Cazzagon et al., 2024). Cases with known etiologies are classified as secondary sclerosing cholangitis which may arise from choledocholithiasis, recurrent infections, trauma, ischemia, toxic exposures, immunologic conditions, and congenital abnormalities, among others (Dyson et al., 2018; Ludwig et al., 2023).

As in PBC, liver transplantation remains the only definitive treatment for advanced PSC with hepatic decompensation (Karlsen et al., 2017; Dyson et al., 2018; Ludwig et al., 2023). No pharmacologic agent has been proven to significantly increase transplant-free survival (Dyson et al., 2018; Hasegawa et al., 2021). As a result, UDCA remains the most commonly prescribed off-label therapy. Low (13–15 mg/kg/day) and intermediate (17–23 mg/kg/day) doses have demonstrated biochemical improvements in alkaline phosphatase (ALP), gamma-glutamyl transferase (GGT), bilirubin, aminotransferases, as well as reduced intestinal ecological disorders and reshaped gut microbiota in IBD (Hasegawa et al., 2021; Cariello et al., 2022; Pan et al., 2024; Manns et al., 2025). High-dose UDCA (≥25 mg/kg/day) by contrast has been associated with increased side effects and is not recommended (Beuers et al., 1992; Manns et al., 2025).

Norucholic acid (NCA), previously known as norUDCA, a side chain–shortened homologue of UDCA, resists taurine and glycine conjugation, allowing for cholehepatic shunting and sustained cycling between hepatocytes and cholangiocytes (Assis and Bowlus, 2023; Bowlus et al., 2023). This promotes bicarbonate-rich, BA-independent bile flow, enhancing choleresis and protecting cholangiocytes with its predominant monomeric secretion augmenting these effects (Fickert et al., 2017; Assis and Bowlus, 2023). NCA also exhibits immunomodulatory activity-suppressing CD8^+^ T cell activation via mTORC1 inhibition and shows antiproliferative and antifibrotic properties in both preclinical and clinical studies (Assis and Bowlus, 2023). A phase II trial reported significant ALP reductions across all tested doses (500–1,500 mg/day), though adverse effects such as pruritus, fatigue, and nasopharyngitis occurred in all groups (Fickert et al., 2017). A phase III trial is underway to further assess long-term safety and efficacy (NCT03872921).

FXR agonists have shown promise in the management of PSC. OCA demonstrated good tolerability at standard dose (5–10 mg/day) in a phase II trial, resulting in significant ALP reduction. Dose-dependent pruritus was reported as the most common side effect (Kowdley et al., 2020). Cilofexor, a nonsteroidal FXR agonist, was evaluated in a 12-week phase II study in non-cirrhotic PSC patients and showed a 21% reduction in ALP at a dose of 100 mg/day (Trauner et al., 2019).

Emerging therapies such as PPAR agonists, IBAT inhibitors, anti-human CCL24 monoclonal CM-101 antibodies and FGF19 analogues are under active investigation. Elafibranor, in the ELMWOOD trial, was well tolerated and led to significant reductions in ALP and stabilization of fibrosis markers over 12 weeks, supporting further investigation of PPAR agonists as disease-modifying agents (Levy et al., 2025a). Similarly, IBAT inhibitors, including volixibat (NCT04663308) and maralixibat (NCT06553768) are in early-phase trials and their efficacy and safety in PSC remain to be determined. CM-101 antibodies (NCT04595825) are also being explored with a phase II trial revealing that dose-dependent (10 mg/kg or 20 mg/kg) treatment was well tolerated and demonstrated anti-inflammatory, anti-fibrotic and anti-cholestatic effects in individuals with PSC compared to placebo. Another phase II trial demonstrated that aldafermin, an engineered FGF19 analogue, potently suppressed hepatic CYP7A1 activity, leading to reduced BA synthesis and significant improvements in non-invasive biomarkers of fibrosis in PSC patients, although it did not significantly reduce ALP levels (Hirschfield et al., 2019).

Given the progressive fibrotic nature of PSC, antifibrotic agents represent a critical area of therapeutic development. Simtuzumab, a monoclonal antibody against lysyl oxidase-like 2 (LOXL2), failed to show efficacy in a phase II trial in PSC, highlighting the complexity of targeting fibrosis in this disease (Muir et al., 2019). Bexotegrast, a αvβ6/αvβ1 integrin inhibitor, demonstrated a favorable safety profile and preliminary efficacy, including antifibrotic effects, liver function improvement, and symptom reduction (NCT04480840), positioning it as a promising candidate.

Considering the multifactorial nature of PSC pathogenesis, future BA-targeted therapies may benefit from combination approaches. For instance, pairing FXR agonists or NCA with antifibrotic agents like bexotegrast could potentially address both cholestatic and fibrotic components of the disease. Understanding how these agents influence BA signaling pathways, transporter expression, and microbiome interactions will be critical to optimizing therapeutic strategies.

#### Intrahepatic cholestasis of pregnancy (ICP)

ICP is the most common pregnancy-specific liver disorder, typically presenting in the second or third trimester. It is characterized by pruritus, elevated liver enzymes, and increased total BA (TBA) levels (Lee et al., 2021; Tang et al., 2023; Tang et al., 2024). Severity is stratified by third-trimester TBA levels: mild ICP is defined as 10–39 μmol/L and severe ICP as ≥ 40 μmol/L (Tang et al., 2024). Although ICP usually resolves postpartum and poses minimal risk to the pregnant individual, it has been associated with increased rates of postpartum hemorrhage, hepatobiliary malignancy, and immune or cardiovascular diseases (Tang et al., 2024). Importantly, it also poses significant fetal risk, including preterm labor, meconium-stained amniotic fluid, and intrauterine fetal demise, particularly when TBA levels exceed 100 μmol/L (Smith and Rood, 2020; Lee et al., 2021; Ovadia et al., 2021). The pathophysiology of ICP remains poorly understood, although evidence suggests that changes in the gut microbiome and genetic predisposition may play a role in its development (Pataia et al., 2017; Tang et al., 2023).

UDCA is the standard first-line, off-label treatment for ICP (Smith and Rood, 2020; Walker et al., 2020; Jiang et al., 2021). Dosing typically ranges from 10–15 mg/kg/day, with escalation up to 21 mg/kg/day in refractory cases (Lee et al., 2021; Kothari et al., 2024). UDCA has been shown to reduce pruritus and improve serum liver tests compared to controls and alternative treatments (Pataia et al., 2017; Walker et al., 2020; Lee et al., 2021). However, its effectiveness in preventing adverse perinatal outcomes remains inconsistent across studies (Kong et al., 2016; Walker et al., 2020; Jiang et al., 2021). UDCA is generally well tolerated by both the mother and fetus with few reported adverse effects beyond nausea and emesis (Kong et al., 2016; Walker et al., 2020). Notably, one study reported enhanced improvements in pruritus and liver parameters when UDCA was co-administered with traditional Chinese medicines such as *A. capillaris Thunb*, *Gardenia*, and *Rhubarb* (Jiang et al., 2021). Further studies are needed to explore the mechanisms and clinical relevance of such combination therapies.

### Pediatric cholestatic diseases

#### Progressive familial intrahepatic cholestasis (PFIC)/Benign recurrent intrahepatic cholestasis (BRIC)

PFIC comprises a group of rare, autosomal recessive cholestatic liver diseases that typically present in infancy or early childhood and are characterized by progressive defects in bile production and transport (Gunaydin and Bozkurter Cil, 2018; Vinayagamoorthy et al., 2021; McKiernan et al., 2023). The main subtypes PFIC1, PFIC2, and PFIC3 result from mutations in genes encoding hepatocanalicular transporters essential for bile formation and flow (van der Woerd et al., 2017). In contrast, BRIC is a milder, non-progressive phenotype caused by mutations in the same genes as PFIC1 and PFIC2, and is characterized by intermittent cholestasis with complete resolution between episodes (van der Woerd et al., 2017).

Mutations in *ATP8B1* (PFIC1/FIC1 deficiency) and *ABCB11* (PFIC2/BSEP deficiency) may cause either BRIC1/BRIC2 or progressive cholestasis with severe pruritus (Baker et al., 2019; Hassan and Hertel, 2022). PFIC2 has an increased risk of hepatocellular carcinoma and cholangiocarcinoma (McKiernan et al., 2023). Mutations in *ABCB4* (PFIC3/MDR3 deficiency) are associated with progressive cholestasis, moderate to severe pruritus, extrahepatic manifestation, and increased risk of hepatobiliary malignancies (Baker et al., 2019; Hassan and Hertel, 2022). If untreated, all PFIC subtypes can progress to fibrosis, cirrhosis, and ESLD (Hassan and Hertel, 2022).

Maralixibat and odevixibat, both IBAT/ASBT inhibitors, are FDA-approved for the treatment of pruritus in PFIC and have shown significant reductions in both pruritus and serum BA levels (Thompson et al., 2022; Miethke et al., 2024). UDCA (10–30 mg/kg/day) remains the off-label drug of choice for PFIC3 and is often used in combination with rifampin and/or cholestyramine to improve bile flow and manage pruritus, though evidence for synergistic benefits is limited (van der Woerd et al., 2017; Gunaydin and Bozkurter Cil, 2018). UDCA is most effective in patients with *ABCB4* missense mutations or MDR3 monoallelic-deficient grafts, while those with nonsense mutations and absent MDR3 expression are typically unresponsive (Gunaydin and Bozkurter Cil, 2018). Selected cases of PFIC1/BRIC1 and PFIC2/BRIC2 may also benefit symptomatically, though evidence remains inconsistent (van der Woerd et al., 2017). Prior to the IBAT inhibitor era, many PFIC1 and PFIC2 patients required surgical interventions, including partial external biliary diversion or liver transplantation (van der Woerd et al., 2017; McKiernan et al., 2023).

Therapeutic strategies for newly identified subtypes–PFIC4, PFIC5, and PFIC6 – are still under investigation. PFIC5, caused by mutations in *NR1H4* encoding FXR, may be a candidate for OCA therapy, although no formal clinical trials have been conducted to date (Beuers et al., 2025). The efficacy of NCA observed in PSC suggests it may also offer enhanced therapeutic benefits across all PFIC and BRIC subtypes, but requires further validation (Stapelbroek et al., 2010).

#### Alagille syndrome (ALGS)

ALGS is a multisystem, autosomal dominant disorder commonly associated with intrahepatic bile duct paucity due to mutations in the *JAG1* or *NOTCH2* genes (Turnpenny and Ellard, 2012; Sutton et al., 2024). These genes encode key components of the Notch signaling pathway, essential for intrahepatic bile duct development (Turnpenny and Ellard, 2012; Kohut et al., 2021; Heinz and Vittorio, 2023; Sutton et al., 2024). Disruption of this pathway can cause a wide range of clinical manifestations, including cholestatic liver disease, cardiac, renal, vascular, skeletal and ocular anomalies, as well as characteristic facial features (Mitchell et al., 2018; Kohut et al., 2021; Ayoub et al., 2023). Diagnosis is often challenging due to variable phenotypes and incomplete penetrance, frequently requiring genetic confirmation (Ayoub et al., 2023). The incidence is estimated at approximately 1 in 30,000 live births (Mitchell et al., 2018; Kohut et al., 2021; Ayoub et al., 2023). While overall survival into adulthood approaches 90%, only 24%–40.3% retain their native liver (Kamath et al., 2020; Ayoub et al., 2023; Vandriel et al., 2023).

Early diagnosis improves native liver survival with genetic testing for *JAG1* and *NOTCH2* mutations being the gold standard (Fligor et al., 2022; Heinz and Vittorio, 2023). However, when genetic testing is unavailable or delayed, liver biopsy combined with syndromic features helps differentiation from biliary atresia (Kohut et al., 2021). Correct diagnosis is critical, as inappropriate Kasai procedures in ALGS patients have been linked to clinical deterioration (Kaye et al., 2010).

The heterogeneous clinical presentation and multisystem involvement of ALGS pose challenges for standardized treatment approach. Effective management requires comprehensive nutritional and medical support alongside pharmacological therapy (Cheng and Rosenthal, 2023). Odevixibat and maralixibat are FDA-approved for severe pruritus in children ≥12 months and ≥3 months of age, respectively. UDCA (10–20 mg/kg/day) has historically been used to manage cholestasis and pruritus, though evidence for efficacy is limited (Kohut et al., 2021; Ayoub and Kamath, 2022). UDCA can be used alone or combined with cholestyramine, rifampin, or naltrexone to improve symptom control (Turnpenny and Ellard, 2012). Liver transplantation remains the definitive treatment for advanced disease.

#### Biliary atresia

Biliary atresia is a progressive inflammatory and fibrosclerosing cholangiopathy that represents the most common cause of neonatal cholestasis and the leading indication for pediatric liver transplantation (Chung et al., 2020; Antala and Taylor, 2022). It is frequently misdiagnosed as ALGS due to overlapping clinical features such as neonatal jaundice with elevated GGT and histological similarities including giant cell hepatitis and ductular proliferation (Kohut et al., 2021; Ayoub and Kamath, 2022). If untreated, biliary atresia rapidly progresses to fibrosis, cirrhosis, and ESLD, with liver transplantation required in patients who do not respond to early surgical intervention (Chung et al., 2020; Antala and Taylor, 2022).

Early surgical treatment with hepatic portoenterostomy (Kasai procedure) within the first 60 days of life is associated with the most favorable long-term outcomes (Heinz and Vittorio, 2023). Post-Kasai, treatment with UDCA (10–15 mg/kg/day) has been shown to significantly improve serum bilirubin clearance and reduce TBA levels compared to controls; however, survival benefit remains unclear (Qiu et al., 2018). In one study of stable post-Kasai patients, UCDA discontinuation led to worsening liver enzyme profiles, which was reversed upon reintroduction. Despite no significant changes in clinical status such as pruritus, bacterial cholangitis, or hepatomegaly, suggesting a beneficial role in maintaining biochemical stability (Willot et al., 2008). By contrast, a retrospective study from Egypt involving 141 infants reported worse outcomes in those treated with UDCA, though this likely reflected the overall poor prognosis of the cohort, with UDCA possibly used as a last resort therapy in severely affected cases (Table 2) (Kotb, 2008).

**TABLE 2:** Therapeutic applications, safety profile, dosage ranges, and regulatory status of current bile acid (BA) combination therapies.

Approval status	Combination/Monotherapy	Therapeutic use	Adverse effects	Typical dosage range	References
Non-BA FDA approved agents	Seladelpar (PPAR-δ agonist) ± UDCA	FDA: PBC Combination: w/UDCA if inadequate response to UDCA alone (PBC) Monotherapy: Cannot tolerate UDCA (PBC)	Headache, abdominal pain, nausea, abdominal distention, dizziness	Adult: 10 mg PO daily ±13–15 mg/kg/day (UDCA)	Abuelazm et al. (2025)
	Elafibranor (PPAR α/δ agonist) ± UDCA	FDA: PBC Combination: w/UDCA if inadequate response to UDCA alone (PBC) Monotherapy: Cannot tolerate UDCA (PBC)	Weight gain/loss, abdominal pain, NVD, arthralgia, constipation, muscle injury, GERD, dry mouth, rash	Adult: 80 mg PO daily ±13–15 mg/kg/day (UDCA)	Ergenc and Heneghan (2025), Levy et al. (2025b)
	Maralixibat (IBAT inhibitor)	FDA: Cholestatic pruritus (PFIC ≥3 months old, ALGS) Off-label: PBC, PSC (investigational)	NVD, abdominal pain, fat-soluble vitamin deficiency, liver test abnormalities, GI bleeding, bone fractures	Adult: 285 mcg/kg PO daily (initial), 570 mcg/kg BID (maintenance) (PFIC) Ped: 190 mcg/kg PO daily (initially), 380 mcg/kg PO daily (maintenance) (ALGS)	Garcia, Hsu and Lin (2023), Miethke et al. (2024)
	Odevixibat (IBAT inhibitor)	FDA: Cholestatic pruritus (PFIC, ALGS) Off-label: PBC, PSC (investigational)	Liver test abnormalities, NVD, abdominal pain, fat-soluble vitamin deficiency	Adult/Ped: 40–120 mcg/kg PO daily (PFIC/ALGS)	Thompson et al. (2022), Ovchinsky et al. (2024)
	Cholestyramine	FDA: Cholestatic pruritus (PBC) Off-label: PFIC, ICP, BA malabsorption syndrome	Constipation, NVD	Adult/Ped: 4–16 g PO daily	Kremer et al. (2008), Patel et al. (2019)
	Resmetirom (THR-β agonist)	FDA: Noncirrhotic MASH w/fibrosis	NVD, pruritus, constipation, abdominal pain, dizziness	Adult: 80 mg PO daily (<100 kg), 100 mg PO daily (>100 kg)	Keam (2024)
	Colesevelam (BA sequestrant)	FDA: T2DM	Constipation, dyspepsia, nausea	Adult: 1,875 mg PO BID daily	Hansen et al. (2017)

This table summarizes the current commonly used indications and investigational therapeutic uses, reported adverse effects, typical dosage ranges for BA-involved combination therapies. References correspond to key supporting literature. NVD = nausea, vomiting, diarrhea.

### Other diseases

#### Metabolic dysfunction–associated steatotic liver disease (MASLD)/Metabolic dysfunction–associated steatohepatitis (MASH)

BA metabolism is markedly disrupted in MASLD and MASH, with characteristic changes in BA synthesis, pool composition, hydrophobicity, and receptor-mediated signaling (Arab et al., 2018). These alterations contribute to steatosis, inflammation, and fibrogenesis, positioning the FXR/FGF19 axis and enterohepatic BA circulation as central therapeutic targets (Simbrunner et al., 2025; Steinberg et al., 2025). Despite strong mechanistic rationale, no BA-directed therapy has yet achieved regulatory approval for MASLD/MASH.

FXR agonists represent the most extensively studied BA-derived approach. OCA demonstrated improvements in hepatic steatosis and fibrosis in early trials, but its development was ultimately discontinued after failing to meet histologic endpoints and due to concerns about pruritus and dyslipidemia in Phase III studies (Neuschwander-Tetri et al., 2015; Younossi et al., 2019). These limitations prompted the advancement of non-steroidal FXR agonists, which were designed to preserve FXR-mediated metabolic and antifibrotic activity while reducing BA-driven adverse effects. Cilofexor produced meaningful reductions in liver fat and aminotransferases, with additive benefits observed in combination studies using firsocostat ± semaglutide (Patel et al., 2020; Alkhouri et al., 2022). Tropifexor similarly reduced liver fat and ALT but remained limited by pruritus and LDL elevations (Sanyal et al., 2023). Additional agents, including vonafexor, TERN-101, and PX-104, have shown early biochemical improvements, whereas EDP-305 and nidufexor were discontinued due to safety concerns and strategic reprioritization, respectively (Fiorucci et al., 2020; Prikhodko et al., 2022; Ratziu et al., 2022; Hu et al., 2024).

Beyond FXR, the BA-responsive receptor TGR5 remains an attractive target. Selective TGR5 agonists such as INT-777 and dual FXR/TGR5 agonists such as INT-767 exhibit potent anti-steatotic and antifibrotic effects in preclinical models (Hodge and Nunez, 2016). However, their clinical translation has been limited by gallbladder filling, cholestasis, and species-dependent receptor expression patterns (Jin et al., 2024).

Additional BA-derived agents have demonstrated potential complementary benefits. NCA has shown improvements in aminotransferases, steatosis, and liver stiffness in Phase II studies, and its histologic efficacy is being further evaluated in the OASIS trial (NCT05083390). Conventional UDCA lowers ALT and GGT but does not significantly improve histology in MASLD/MASH (Lin et al., 2022). TUDCA has demonstrated robust cytoprotective effects in preclinical settings, though these findings have not been confirmed in controlled clinical trials (Wang et al., 2024).

Aramchol, a bile acid–fatty acid conjugate that inhibits stearoyl-CoA desaturase 1 (SCD1), has shown meaningful reductions in liver fat and favorable metabolic effects in Phase II studies (Safadi et al., 2014). Although the ARREST Phase IIb trial did not meet its primary endpoint, trends toward MASH resolution, fibrosis improvement, and improved liver enzymes supported its advancement into the Phase III ARMOR program (Ratziu et al., 2021). In this ongoing trial, the optimized 300 mg BID dose has demonstrated promising antifibrotic activity based on conventional histology, paired-ranked reads, and AI-assisted digital pathology (Ratziu et al., 2025).

Aldafermin, an engineered analogue of FGF19, leads to rapid and substantial reductions in liver fat and improvements in circulating biomarkers of steatohepatitis and fibrosis (Harrison et al., 2021). Although it did not meet its primary histologic endpoint in patients with compensated cirrhosis, consistent improvements in non-invasive fibrosis measures were observed (Rinella et al., 2024). Because of theoretical risks related to FGF19-associated mitogenicity, long-term monitoring remains important (Ursic-Bedoya et al., 2024).

Agents that modulate enterohepatic BA circulation offer a more indirect therapeutic approach. ASBT/IBAT inhibitors reduce BA pool size and hepatic exposure, but the most advanced agent, volixibat, failed to meet prespecified MRI-PDFF and ALT efficacy thresholds and was discontinued (Salic et al., 2019; Newsome et al., 2020). Additional approaches, including BA sequestrants and microbiome-targeted therapies that reshape BA composition and influence FXR/TGR5 signaling, have shown encouraging effects in preclinical models but have not yet been studied in MASLD/MASH clinical trials (Takahashi et al., 2020; Duarte et al., 2025).

Non BA-directed therapies highlight the need for multi-pathway approaches in MASH. The THR-β agonist resmetirom, now FDA-approved for noncirrhotic MASH with fibrosis, produces substantial reductions in liver fat and steatohepatitis (Harrison et al., 2024; Targher et al., 2025). Additional late-stage investigational candidates include the GLP-1 receptor agonist semaglutide, the pan-PPAR agonist lanifibranor, the FGF21 analogues efruxifermin and pegozafermin, the fatty acid synthase inhibitor denifanstat, and survodutide, a dual glucagon/GLP-1 receptor agonist (Israelsen et al., 2024). Together, these therapies reflect the complex metabolic, inflammatory, and fibrotic pathways underlying MASH and support the rationale for combination strategies that integrate BA-directed, metabolic, and antifibrotic mechanisms.

### Type 2 diabetes mellitus (T2DM)

T2DM is the common, insulin-resistant variant of diabetes mellitus that is commonly associated with obesity, fatty liver disease, cardiovascular issues and presents a major crisis for human health (Nauck et al., 2021). As more research is being conducted on the role of BAs in insulin signaling, energy metabolism and glucose tolerance, BAs have become a promising therapeutic target in the treatment of T2DM.

BA sequestrants, such as colesevelam, are FDA-approved for T2DM and have demonstrated clinically meaningful reductions in HbA1c in meta-analyses of randomized controlled trials, with additional benefits for lipid profiles (Sonne et al., 2014; Hansen et al., 2017). The mechanism is thought to involve modulation of BA signaling through FXR and TGR5, which impacts glucose metabolism and GLP-1 secretion (Sonne et al., 2014).

Novel BA derivatives, such as berberine ursodeoxycholate (HTD1801), have shown promise in recent phase II randomized clinical trials (NCT06411275). HTD1801, an ionic salt of berberine and UDCA, led to significant reductions in HbA1c and improvements in glycemic, cardiometabolic, and liver-related parameters over 12 weeks, with a favorable safety profile (Ji et al., 2025). These effects are attributed to AMP kinase activation, NLRP3 inflammasome inhibition, and improved insulin sensitivity (Ji et al., 2025).

Another recent study identified MABAs as promising clinical markers of glycemic control (Lin et al., 2025). Tryptophan-conjugated CA (Trp-CA) was markedly reduced in patients with T2DM compared with healthy controls, and its administration improved glucose tolerance in diabetic mice (Lin et al., 2025). The GPCR MRGPRE was subsequently identified as the receptor mediating these metabolic effects (Lin et al., 2025). These findings suggest that Trp-CA–MRGPRE signaling may represent a novel therapeutic avenue for T2DM.

Ongoing clinical trials are evaluating additional BA-based therapies, including FXR and TGR5 agonists, and agents that modulate BA transport. Multiple trials on the efficacy and safety of UDCA in T2DM (NCT01337440, NCT05416580, NCT02033876) revealed that treatment resulted in a reduction of HbA1c levels, increased GLP-1 secretion, glucose homeostasis and showed beneficial effects on metabolic and oxidative stress parameters (Shima et al., 2018; Calderon et al., 2020; Lakić et al., 2024). Additional studies (NCT05902468) further exploring UDCA’s therapeutic potential in T2DM are currently underway.

Trials on TUDCA (NCT00771901, NCT03331432) revealed improvements to insulin action and vascular function in participants through the modulation of obesity-related insulin resistance and inhibition of endoplasmic reticulum stress (Amen et al., 2019). A phase II study of OCA (NCT00501592) revealed that a 25–50 mg treatment over 6 weeks led to improved insulin sensitivity, minor loss of body weight and enhanced FGF19 levels in the serum in patients with T2DM and MASLD (Mudaliar et al., 2013). These approaches aim to leverage the hormonal and signaling properties of BAs to improve glucose homeostasis, insulin sensitivity, and reduce inflammation.

### Cerebrotendinous xanthomatosis (CTX)

CTX is a rare, autosomal recessive disorder of BA synthesis and lipid storage caused by pathogenic mutation to the CYP27A1 gene (DeBarber and Duell, 2021; Islam, Hoggard and Hadjivassiliou, 2021). This leads to reduced levels of primary BAs, particularly CDCA, which in turn disrupts the normal negative-feedback regulation of CYP7A1, allowing for a toxic build up of intermediate metabolites, such as cholestanol, to accumulate in various tissues (Mandia et al., 2019; DeBarber and Duell, 2021; Duell et al., 2023). These deposits commonly aggregate in the central nervous system (CNS), namely the cerebellum, as well as tendons and the lenses of the eyes (Islam et al., 2021). Disease progression can manifest into severe neurocognitive impairment, Parkinsonism, peripheral neuropathy, cerebellar ataxia, dementia, behavioral and psychiatric disturbances, seizures, chronic diarrhea, early onset cataracts and tendon xanthomata if left untreated (Islam et al., 2021; Nóbrega et al., 2022; Kisanuki et al., 2025).

The FDA and European Medicines Agency have approved CDCA for treatment of CTX in adults (Bouwhuis et al., 2023; Jalal et al., 2025). Administration of 250 mg PO TID is generally well tolerated and has been reported to improve neurologic manifestations, slow disease progression and decrease all CTX biomarkers (Nóbrega et al., 2022; Kisanuki et al., 2025). A randomized CDCA withdrawal study on patients with CTX revealed significant increases in metabolite biomarkers when treatment with CDCA was not administered, with 61% of placebo participants requiring rescue medication (Kisanuki et al., 2025). Despite this, around 20% of patients who are administered CDCA for CTX continue to show signs of deterioration, in which case, CA may be utilized as an adjunctive or alternative therapy (Mandia et al., 2019; Nóbrega et al., 2022). Efficacy of CA is noted to significantly reduce cholestanol levels in all patients with no adverse effects reported in one study (Mandia et al., 2019). CDCA treatment does not typically reduce tendon xanthomas nor improve cataracts in patients with CTX and is currently contraindicated for use during pregnancy (Nóbrega et al., 2022). However, one case series of 19 pregnancies noted that no complications were reported in newborns delivered by CDCA-treated birth parents postterm (Duell et al., 2023; Zaccai et al., 2024). CDCA is not currently FDA-approved for usage in pediatric individuals although the current recommended dosing is 5–15 mg/kg/day (Table 3) (Nóbrega et al., 2022).

**TABLE 3:** Cholestatic and non-cholestatic disease prevalence profile, recommended therapeutic bile acid (BA) agent and efficacy.

Categorization	Disease	Prevalence	BAs/BA derivatives used	Reported efficacy	References
Adult cholestatic disorder	Primary biliary cholangitis (PBC)	∼1.9–40.9 per 100,000	UDCA, OCA (second-line)	UDCA improves transplant-free survival; OCA effective in UDCA non-responders	Levy et al. (2025a)
	Primary sclerosing cholangitis (PSC)	∼11.6 per 100,000	UDCA, OCA, NCA (investigational)	Mixed results; UDCA improves biochemistry but no survival benefit; NCA promising in trials	Cooper et al. (2022)
	Intrahepatic cholestasis of pregnancy (ICP)	∼200–300 per 100,000 pregnancies	UDCA	Effective for maternal symptom relief and biochemical improvement; fetal outcome benefit uncertain	Sadeghi (2024)
Pediatric cholestatic disorder	Progressive familial intrahepatic cholestasis (PFIC)	∼1 per 50,000–100,000	UDCA (Particularly PFIC3)	Minimal in PFIC1/2; beneficial in PFIC3 with select genotypes (e.g., missense ABCB4)	Mighiu et al. (2022)
	Benign recurrent intrahepatic cholestasis (BRIC)	∼1 per 50,000–100,000	UDCA	Symptom relief in some patients; variable response between episodes	Daniel et al. (2022)
	Alagille syndrome	∼3 per 100,000 live births	UDCA	Limited efficacy; may reduce pruritus and improve biochemistry in some cases	Kohut et al. (2021)
	Biliary atresia	∼5 per 100,000 live births	UDCA	Adjunctive post-Kasai; improves biochemistry but unclear survival benefit	Hartley et al. (2009)
Other diseases	Metabolic dysfunction–associated steatotic liver disease (MASLD)/Metabolic dysfunction–associated steatohepatitis (MASH)	∼15,000 per 100,000	UDCA, OCA (investigational)	Limited efficacy, UDCA lowers ALT and GGT but not histology, OCA failed to meet histological endpoints in phase III trials	Huang et al. (2025)
	Inflammatory bowel disease (IBD)	∼11 per 100,000	UDCA	Limited efficacy; reshapes the gut microbiome	Lewis et al. (2023), Caron et al. (2024)
	Cerebrotendinous xanthomatosis (CTX)	∼1 per 75,000–150,000	CDCA	Effective in controlling abnormal biomarkers and avoiding disease progression	Mandia et al. (2019), Kisanuki et al. (2025)
	Type 2 diabetes mellitus (T2DM)	∼6,000–7,000 per 100,000	UDCA, TUDCA, OCA (investigational)	Limited efficacy; may help to control glucose metabolism and support altered BA profile	Khan et al. (2020)

This table summarizes the approximate prevalence of the various cholestatic and non-cholestatic disorders. Efficacy is provided based on clinical trial data from the listed BAs.

## Future considerations

The therapeutic potential of BAs extends far beyond classical roles in hepatic and metabolic regulations. Two particularly promising areas for future exploration are the neuroprotective properties of hydrophilic BAs and the expanding use of BA-stabilized vesicular systems (bilosomes) for targeted drug and vaccine delivery.

### BAs in neuroprotection

Hydrophilic BAs, notably UDCA and its conjugates GUDCA and TUDCA, are increasingly recognized for their neuroprotective properties in models of neurological, neurodegenerative, and neuropsychiatric diseases (Zangerolamo et al., 2021; Huang et al., 2022a; Khalaf et al., 2022). Conjugated BAs require active transport into the brain although lipophilic, unconjugated BAs, such as CDCA, are capable of crossing the BBB from peripheral circulation through passive diffusion and exert cytoprotective effects through multiple mechanisms (Mulak, 2021; Zangerolamo et al., 2021; Huang et al., 2022a; Khalaf et al., 2022; Xing et al., 2023). Additionally, they serve as chemical chaperones that support proper protein folding and stability, which is crucial in diseases involving protein misfolding and aggregation (Zangerolamo et al., 2021; Khalaf et al., 2022; Xing et al., 2023).

Preclinical studies demonstrate that UDCA reduces neuronal apoptosis, ROS, and pro-inflammatory cytokine production in models of neurodegeneration, while GUDCA and TUDCA exhibit similar neuroprotective effects across neurological and neuropsychiatric models (Zangerolamo et al., 2021; Huang et al., 2022a). Their therapeutic potential appears to be disease-specific. For instance, all three have shown benefit in Huntington’s disease models, while UDCA and TUDCA are more effective in Parkinson’s (PD) and Alzheimer’s disease (AD) models, and GUDCA in bilirubin encephalopathy (Huang et al., 2022a).

Beyond their direct neuroprotective actions, BAs influence the microbiota-gut–brain axis. Recent animal studies demonstrate that disruptions in the gut microbiota can actively contribute to neurodegenerative disease by reshaping and promoting microglial behavior and activation which, in conjunction with neuroinflammatory responses, are central features of many neurodegenerative disorders (Sampson et al., 2016; Burberry et al., 2020; Wilson et al., 2023; Loh et al., 2024). Although microglial cells exhibit phagocytic properties to eliminate protein aggregates in the brain, excessive uptake of such compounds can lead to neuroinflammation, phagocytic impairment and eventual neurodegeneration (Gao et al., 2023). Because microbial metabolites and signaling pathways exert powerful control over glial activity, the microbiota–gut–brain axis has emerged as a compelling therapeutic target for slowing or modifying the course of neurodegeneration (Cook and Prinz, 2022; Loh et al., 2024).

Importantly, gut microbes modify BA composition through biotransformation, while BAs shape the gut microbial community. Disturbances to bacterial BS hydrolases, which generate deconjugated BAs that are less toxic to the gut microbiota, reshape the BA profile in both the serum and brain, ultimately affecting signaling within the CNS (Wu et al., 2024a). These bidirectional interactions regulate metabolic and signaling pathways that impact neuroinflammation and immune responses relevant to neurodegeneration, and CNS health (Mulak, 2021; Wang et al., 2023; Wu et al., 2024b). Alterations in BA profiles and gut microbiota composition have been observed in AD and underscored by the aforementioned CTX, implicating GLBA crosstalk in neurodegeneration pathophysiology (Mulak, 2021; Wang et al., 2023; Wu et al., 2024b).

In AD, amyloid β (Aβ) plaques and Tau protein aggregates within neurons remain the most recognized biomarkers of disease progression (Wu et al., 2024b). Aβ exposure has been found to activate the early stress c-Jun N-terminal kinase (JNK) pathway which eventually induces apoptosis (Viana et al., 2010). Microtubule affinity–regulating kinase (MARK4), whose overexpression in AD induces synaptic and dendritic spine defects, is also associated with increases in Tau phosphorylation, leading to aggregates (Anwar et al., 2020; Wu et al., 2024b). In terms of BA profiles, LCA-CA and DCA-CA ratios are significantly elevated in reporting studies, with microbiota-derived glyco-DCA/LCA and tauro-LCA also showing significant increases and being associated with reduced cognitive outcomes in patients with AD (Pan et al., 2017; MahmoudianDehkordi et al., 2019).

Therapeutically, CA has been shown to inhibit the Tau phosphorylation activity of MARK4, thereby reducing cognitive deficits and neuronal lesions (Anwar et al., 2020; Wu et al., 2024b). Other MARK4 inhibitors such as donepezil and rivastigmine tartrate are currently undergoing research to explore similar therapeutic potential with promising results (Shamsi et al., 2020). TUDCA attenuates Aβ toxicity by dampening JNK-driven stress signaling, stabilizing mitochondrial membranes, and limiting activation of pro-apoptotic cascades (Wu et al., 2024b). Because clinical antibody therapies against Aβ have shown only modest benefit, these BA-mediated mechanisms offer a complementary therapeutic avenue targeting both Aβ and Tau pathology (Asher and Priefer, 2022). A current phase II trial is underway to evaluate the safety and efficacy of a combination therapy of TUDCA and sodium phenylbutyrate in patients with AD (NCT03533257).

PD by contrast, is a neurodegenerative condition characterized by the progressive loss of catecholamines and cholinergic neurons in conjunction with Lewy body formations composed of aggregated α-synuclein deposits (Hurley et al., 2022). Both UDCA and TUDCA have been observed to inhibit neuroinflammation and reduce glial activity in PD mouse models (Loh et al., 2024). Two recent clinical trials (NCT02967250, NCT03840005) that further evaluate the efficacy of UDCA in PD found that high-dose administration (30 mg/kg/day) was well tolerated in early PD (Payne et al., 2023). Treatment with dioscin, a TGR5 agonist, displayed increased GLP-1 activation and improvements to motor function in mouse models (Sun et al., 2021; Mao et al., 2023; Loh et al., 2024). GLP-1 activity enhances insulin secretion, lowering serum levels of glucose which lead to reduced levels of glial activity (Sun et al., 2021; Mao et al., 2023; Nowell et al., 2023). Co-administration of UDCA and dioscin was shown to enhance the neuroprotective properties of either drug by itself.

Several studies report elevated circulating secondary BAs such as DCA, LCA, and HDCA, and variable but notable changes in primary BAs (Yakhine-Diop et al., 2020; Li et al., 2021; Shao et al., 2021; Hurley et al., 2022). Some studies identify increased plasma CA and elevated glycine- or taurine-conjugated primary BAs, whereas others report reduced glycocholic acid and glycochenodeoxycholic acid in the frontal cortex, highlighting tissue-specific differences that may reflect distinct pathogenic mechanisms (Yakhine-Diop et al., 2020; Shao et al., 2021; Hurley et al., 2022; Kalecký and Bottiglieri, 2023). PD patients and prodromal PD mouse models also show reduced levels of the neuroprotective BAs UDCA and TUDCA, suggesting impaired BA-mediated cytoprotection (Graham et al., 2018; Shao et al., 2021). Additional evidence, including the increased PD risk after cholecystectomy and secondary BA elevations following gut microbiota transfer, supports a role for altered BA signaling within the GLBA in early PD pathogenesis (Kim et al., 2021; Xu et al., 2023).

Despite promising preclinical data, further research is needed to elucidate the molecular mechanisms of BA signaling within the CNS, optimize pharmacokinetics and dosing strategies, and establish efficacy and safety through large-scale clinical trials. Harnessing the neuroprotective and immunomodulatory effects of BAs may open new avenues for treating neurodegenerative and neuropsychiatric disorders, particularly when integrated with targeted delivery systems such as bilosomes.

### BA-based nanocarriers

Bilosomes are BA-stabilized vesicular systems composed of non-ionic surfactants (niosomes), phospholipids (liposomes), and amphiphilic BSs, which confer superior stability, deformability, and protection against enzymatic and acidic degradation compared to conventional liposomes and niosomes (Mitrović et al., 2025). The integration of BSs into the bilayer enhances vesicle integrity in the gastrointestinal tract, promotes mucosal penetration, and facilitates absorption, resulting in increased oral bioavailability of peptides, proteins, and other labile molecules (Aburahma, 2016; Nayak et al., 2023; Mitrović et al., 2025).

Bilosome-based delivery systems demonstrate remarkable versatility across multiple non-invasive administration routes. Surface-modification of bilosomes enhance cell surface receptor identification and ligand detection to allow for improved targeting efficiency and drug delivery to a diverse number of pathologies (Nayak et al., 2023; Priya et al., 2023; Mitrović et al., 2025). In oral drug delivery, bilosomes protect encapsulated agents from gastrointestinal deposition and enhance intestinal absorption (Aburahma, 2016; Nayak et al., 2023; Mitrović et al., 2025). For oral vaccines, bilosomes facilitate efficient antigen uptake via Peyer’s patches, leading to robust mucosal and systemic immune responses, with efficacy demonstrated in preclinical and early clinical studies (Wilkhu et al., 2013; Shukla et al., 2016). For transdermal administration, the ultra-deformable properties of bilosomes allow traversal of the stratum corneum, significantly improving skin penetration and drug deposition (Nayak et al., 2023; Mitrović et al., 2025). In ocular applications, bilosomes enhance corneal permeation, prolong retention time, and improve stability, resulting in better therapeutic availability for ophthalmic conditions (Nayak et al., 2023; Rajbhar et al., 2025). Intranasally, bilosomes protect encapsulated agents from enzymatic degradation, increase mucosal adhesion, and facilitate direct nose-to-brain transport, offering a promising strategy for CNS-targeted therapies and vaccines (Mitrović et al., 2025).

In the context of hepatobiliary diseases, bilosome-based formulations have shown promise in improving the delivery and therapeutic efficacy of agents such as daclatasvir, sofosbuvir, silymarin, and pitavastatin, as well as bioactive polysaccharides th anti-hepatocarcinogenic activity (Matloub et al., 2018; Joseph Naguib et al., 2020; El-Nabarawi et al., 2021). Studies demonstrate that bilosome encapsulation can enhance hepatic targeting, increase drug bioavailability in the liver, and improve pharmacodynamic outcomes in models hepatitis C and hepatocellular carcinoma (Matloub et al., 2018; Joseph Naguib et al., 2020; El-Nabarawi et al., 2021).

**FIGURE 5:**
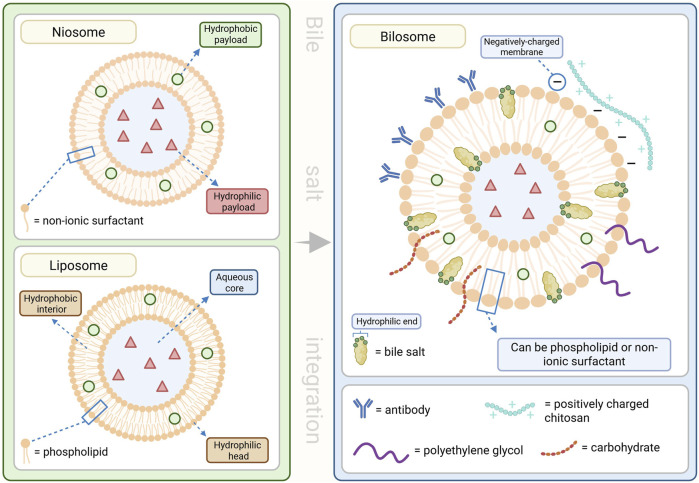
Structures of lipid nanocarriers and various bilosome surface-modifications. Lipid nanocarriers are capable of transporting both hydrophilic and hydrophobic materials through a variety of administration routes. Bile salt (BS) integrated lipid nanocarriers provide improved stability and efficiency with surface-modifications that enhance these delivery mechanisms. This figure was created in BioRender.com.

Recent advancements include probilosomes and surface-modified bilosomes, which offer improved targeting specificity, formulation stability, and delivery efficiency (Nayak et al., 2023; Rajbhar et al., 2025). These platforms enable incorporation of targeting ligands, including antibodies, carbohydrates or peptides to optimize tissue-specific uptake and therapeutic outcomes (Nayak et al., 2023; Priya et al., 2023; Mitrović et al., 2025; Rajbhar et al., 2025). Polymer coatings such as the positively charged chitosan interact with the negatively charged surface of the bilosome to improve mucoadhesion and protect the vesicles in the gastrointestinal environment (Nayak et al., 2023; Priya et al., 2023; Mitrović et al., 2025). Polyethylene glycol integration increases circulation time and reduces immune recognition while also improving the ability to bypass the first-pass effect and avoid rapid metabolism (Figure 5) (Nayak et al., 2023; Mitrović et al., 2025).

While no bilosome-based therapies have yet received regulatory approval for clinical use, ongoing research is addressing formulation optimization, large-scale manufacturing, and long-term safety. These efforts aim to facilitate clinical translation and expand therapeutic options for diverse diseases.

## Conclusion

BAs, once regarded primarily as digestive agents, are now recognized as vital regulators of metabolism, immune function, and cell signaling, with expanding therapeutic potential in liver, metabolic, and neurological disorders. Their interactions with receptors such as FXR, VDR and TGR5 along with their cytoprotective and anti-inflammatory properties, points to a wide range of promising treatments including the modulation of the gut microbiota, anti-inflammatory and anti-pruritic therapies, neuroprotective and metabolic signaling and regulation implications and drug delivery potential as a nanocarrier. Continued research is needed to determine their therapeutic efficacy across disease contexts, refine delivery systems such as bilosomes, and confirm safety and effectiveness through well designed clinical studies.

## References

[B1] Abuelazm M. Alsakarneh S. Tanashat M. Manasrah A. Ibrahim A. A. Parajuli S. (2025). The efficacy and safety of seladelpar for primary biliary cholangitis: a systematic review and meta‐analysis. JGH Open An Open Access J. Gastroenterology Hepatology 9 (9), e70265. 10.1002/jgh3.70265 40893425 PMC12394184

[B2] Aburahma M. H. (2016). Bile salts-containing vesicles: promising pharmaceutical carriers for oral delivery of poorly water-soluble drugs and peptide/protein-based therapeutics or vaccines. Drug Deliv. 23 (6), 1847–1867. 10.3109/10717544.2014.976892 25390191

[B3] Adachi R. Honma Y. Masuno H. Kawana K. Shimomura I. Yamada S. (2005). Selective activation of vitamin D receptor by lithocholic acid acetate, a bile acid derivative. J. Lipid Res. 46 (1), 46–57. 10.1194/jlr.M400294-JLR200 15489543

[B4] Alkhouri N. Herring R. Kabler H. Kayali Z. Hassanein T. Kohli A. (2022). Safety and efficacy of combination therapy with semaglutide, cilofexor and firsocostat in patients with non-alcoholic steatohepatitis: a randomised, open-label phase II trial. J. Hepatology 77 (3), 607–618. 10.1016/j.jhep.2022.04.003 35439567

[B5] Alnouti Y. (2009). Bile acid sulfation: a pathway of bile acid elimination and detoxification. Toxicol. Sci. An Official J. Soc. Toxicol. 108 (2), 225–246. 10.1093/toxsci/kfn268 19131563

[B6] Amaral J. D. Viana R. J. S. Ramalho R. M. Steer C. J. Rodrigues C. M. P. (2009). Bile acids: regulation of apoptosis by ursodeoxycholic acid. J. Lipid Res. 50 (9), 1721–1734. 10.1194/jlr.R900011-JLR200 19417220 PMC2724780

[B7] Amen O. M. Sarker S. D. Ghildyal R. Arya A. (2019). Endoplasmic reticulum stress activates unfolded protein response signaling and mediates inflammation, obesity, and cardiac dysfunction: therapeutic and molecular approach. Front. Pharmacol. 10, 977. 10.3389/fphar.2019.00977 31551782 PMC6747043

[B8] Antala S. Taylor S. A. (2022). Biliary atresia in children: update on disease mechanism, therapies, and patient outcomes. Clin. Liver Disease 26 (3), 341–354. 10.1016/j.cld.2022.03.001 35868678 PMC9309872

[B9] Anwar S. Shamsi A. Kar R. K. Queen A. Islam A. Ahmad F. (2020). Structural and biochemical investigation of MARK4 inhibitory potential of cholic acid: towards therapeutic implications in neurodegenerative diseases. Int. J. Biol. Macromol. 161, 596–604. 10.1016/j.ijbiomac.2020.06.078 32535203

[B10] Arab J. P. Arrese M. Trauner M. (2018). Recent insights into the pathogenesis of nonalcoholic fatty liver disease. Annu. Rev. Pathology 13, 321–350. 10.1146/annurev-pathol-020117-043617 29414249

[B11] Asher S. Priefer R. (2022). Alzheimer’s disease failed clinical trials. Life Sci. 306, 120861. 10.1016/j.lfs.2022.120861 35932841

[B12] Assis D. N. Bowlus C. L. (2023). Recent advances in the management of primary sclerosing cholangitis. Clin. Gastroenterology Hepatology 21 (8), 2065–2075. 10.1016/j.cgh.2023.04.004 37084929

[B13] Ayoub M. D. Kamath B. M. (2022). Alagille syndrome: current understanding of pathogenesis, and challenges in diagnosis and management. Clin. Liver Dis. 26 (3), 355–370. 10.1016/j.cld.2022.03.002 35868679

[B14] Ayoub M. D. Bakhsh A. A. Vandriel S. M. Keitel V. Kamath B. M. (2023). Management of adults with alagille syndrome. Hepatol. Int. 17 (5), 1098–1112. 10.1007/s12072-023-10578-x 37584849 PMC10522532

[B15] Bai S. H. Chandnani A. Cao S. (2024). Bile acids in inflammatory bowel disease: from pathophysiology to treatment. Biomedicines 12 (12), 2910. 10.3390/biomedicines12122910 39767816 PMC11673883

[B16] Baker A. Kerkar N. Todorova L. Kamath B. M. Houwen R. H. J. (2019). Systematic review of progressive familial intrahepatic cholestasis. Clin. Res. Hepatology Gastroenterology 43 (1), 20–36. 10.1016/j.clinre.2018.07.010 30236549

[B17] Bedke T. Stumme F. Tomczak M. Steglich B. Jia R. Bohmann S. (2024). Protective function of sclerosing cholangitis on IBD. Gut 73 (8), 1292–1301. 10.1136/gutjnl-2023-330856 38839272 PMC11287650

[B18] Beuers U. Spengler U. Kruis W. Aydemir U. Wiebecke B. Heldwein W. (1992). Ursodeoxycholic acid for treatment of primary sclerosing cholangitis: a placebo-controlled trial. Hepatol. Baltim. Md 16 (3), 707–714. 10.1002/hep.1840160315 1505913

[B19] Beuers U. Kremer A. E. Bolier R. Elferink R. P. J. O. (2014). Pruritus in cholestasis: facts and fiction. Hepatology 60 (1), 399–407. 10.1002/hep.26909 24807046

[B20] Beuers U. Wolters F. Oude Elferink R. P. J. (2023). Mechanisms of pruritus in cholestasis: understanding and treating the itch. Nat. Rev. Gastroenterology and Hepatology 20 (1), 26–36. 10.1038/s41575-022-00687-7 36307649

[B21] Beuers U. Banales J. M. Karpen S. J. Keitel V. Williamson C. Trauner M. (2025). History and prospects of bile acid therapies. J. Hepatology 83, 1172–1188. [Preprint]. 10.1016/j.jhep.2025.06.010 40545045 PMC13094352

[B22] Bouwhuis N. Jacobs B. A. W. Kemper E. M. (2023). Product development and quality of pharmacy compounded chenodeoxycholic acid capsules for Dutch cerebrotendinous xanthomatosis patients. Front. Pharmacol. 14, 1264997. 10.3389/fphar.2023.1264997 37915417 PMC10616945

[B23] Bowlus C. L. Arrivé L. Bergquist A. Deneau M. Forman L. Ilyas S. I. (2023). AASLD practice guidance on primary sclerosing cholangitis and cholangiocarcinoma. Hepatol. Baltim. Md 77 (2), 659–702. 10.1002/hep.32771 36083140

[B24] Burberry A. Wells M. F. Limone F. Couto A. Smith K. S. Keaney J. (2020). C9orf72 suppresses systemic and neural inflammation induced by gut bacteria. Nature 582 (7810), 89–94. 10.1038/s41586-020-2288-7 32483373 PMC7416879

[B25] Cabrera D. Arab J. P. Arrese M. (2019). UDCA, NorUDCA, and TUDCA in liver diseases: a review of their mechanisms of action and clinical applications. Handb. Exp. Pharmacol. 256, 237–264. 10.1007/164_2019_241 31236688

[B26] Cadena Sandoval M. Haeusler R. A. (2025). Bile acid metabolism in type 2 diabetes mellitus. Nat. Rev. Endocrinol. 21 (4), 203–213. 10.1038/s41574-024-01067-8 39757322 PMC12053743

[B27] Calderon G. McRae A. Rievaj J. Davis J. Zandvakili I. Linker-Nord S. (2020). Ileo-colonic delivery of conjugated bile acids improves glucose homeostasis *via* colonic GLP-1-producing enteroendocrine cells in human obesity and diabetes. EBioMedicine 55, 102759. 10.1016/j.ebiom.2020.102759 32344198 PMC7186521

[B28] Cariello M. Gadaleta R. M. Moschetta A. (2022). The gut-liver axis in cholangiopathies: focus on bile acid based pharmacological treatment. Curr. Opin. Gastroenterology 38 (2), 136–143. 10.1097/MOG.0000000000000807 35034082 PMC10826921

[B29] Caron B. Honap S. Peyrin-Biroulet L. (2024). Epidemiology of inflammatory bowel disease across the ages in the era of advanced therapies. J. Crohn’s and Colitis 18 (Suppl. 2), ii3–ii15. 10.1093/ecco-jcc/jjae082 39475082 PMC11522978

[B30] Catz S. D. Johnson J. L. (2001). Transcriptional regulation of bcl-2 by nuclear factor κB and its significance in prostate cancer. Oncogene 20 (50), 7342–7351. 10.1038/sj.onc.1204926 11704864

[B31] Cazzagon N. Sarcognato S. Catanzaro E. Bonaiuto E. Peviani M. Pezzato F. (2024). Primary sclerosing cholangitis: diagnostic criteria. Tomography 10 (1), 47–65. 10.3390/tomography10010005 38250951 PMC10820917

[B32] Chen B. Bai Y. Tong F. Yan J. Zhang R. Zhong Y. (2023). Glycoursodeoxycholic acid regulates bile acids level and alters gut microbiota and glycolipid metabolism to attenuate diabetes. Gut Microbes 15 (1), 2192155. 10.1080/19490976.2023.2192155 36967529 PMC10054359

[B33] Cheng K. Rosenthal P. (2023). Diagnosis and management of alagille and progressive familial intrahepatic cholestasis. Hepatol. Commun. 7 (12), e0314. 10.1097/HC9.0000000000000314 38055640 PMC10984671

[B34] Cheng J. Fang Z. Z. Kim J. H. Krausz K. W. Tanaka N. Chiang J. Y. L. (2014). Intestinal CYP3A4 protects against lithocholic acid-induced hepatotoxicity in intestine-specific VDR-Deficient mice. J. Lipid Res. 55 (3), 455–465. 10.1194/jlr.M044420 24343899 PMC3934730

[B35] Chiang J. Y. L. (2009). Bile acids: regulation of synthesis: thematic review series: bile acids. J. Lipid Res. 50 (10), 1955–1966. 10.1194/jlr.R900010-JLR200 19346330 PMC2739756

[B36] Chiang J. Y. L. (2013). Bile acid metabolism and signaling. Compr. Physiol. 3 (3), 1191–1212. 10.1002/cphy.c120023 23897684 PMC4422175

[B37] Chiang J. Y. L. Ferrell J. M. (2020). Bile acid receptors FXR and TGR5 signaling in fatty liver diseases and therapy. Am. J. Physiology. Gastrointest. Liver Physiology 318 (3), G554–G573. 10.1152/ajpgi.00223.2019 31984784 PMC7099488

[B38] Chung P. H. Y. Zheng S. Tam P. K. H. (2020). Biliary atresia: east *versus* west. Seminars Pediatr. Surg. 29 (4), 150950. 10.1016/j.sempedsurg.2020.150950 32861448

[B39] Collins S. L. Stine J. G. Bisanz J. E. Okafor C. D. Patterson A. D. (2023). Bile acids and the gut microbiota: metabolic interactions and impacts on disease. Nat. Rev. Microbiol. 21 (4), 236–247. 10.1038/s41579-022-00805-x 36253479 PMC12536349

[B40] Cook J. Prinz M. (2022). Regulation of microglial physiology by the microbiota. Gut Microbes 14 (1), 2125739. 10.1080/19490976.2022.2125739 36151874 PMC9519021

[B41] Cooper J. Markovinovic A. Coward S. Shaheen A. Swain M. Panaccione R. (2022). Incidence and prevalence of primary sclerosing cholangitis: a meta-analysis of population-based studies. Inflamm. Bowel Dis. 28 (Suppl. ment_1), S46–S47. 10.1093/ibd/izac015.072 PMC11532590 38052097

[B42] Daniel T. Fung P. Chen H.-W. (2022). S2931 benign recurrent intrahepatic cholestasis (BRIC) managed with plasmapheresis. Official Journal Am. Coll. Gastroenterology | ACG 117 (10S), e1905–e1906. 10.14309/01.ajg.0000868364.31120.0e

[B43] DeBarber A. E. Duell P. B. (2021). Update on cerebrotendinous xanthomatosis. Curr. Opin. Lipidol. 32 (2), 123–131. 10.1097/MOL.0000000000000740 33630770

[B44] di Gregorio M. C. Cautela J. Galantini L. (2021). Physiology and physical chemistry of bile acids. Int. J. Mol. Sci. 22 (4), 1780. 10.3390/ijms22041780 33579036 PMC7916809

[B45] Dong B. Zhou Y. Wang W. Scott J. Kim K. Sun Z. (2020). Vitamin D receptor activation in liver macrophages ameliorates hepatic inflammation, steatosis, and insulin resistance in mice. Hepatology 71 (5), 1559–1574. 10.1002/hep.30937 31506976

[B46] Duarte L. Magne F. Gotteland M. (2025). Gut microbiota in patients with metabolic, dysfunction-associated steatotic liver disease. Curr. Opin. Clin. Nutr. Metabolic Care 28 (4), 307–315. 10.1097/MCO.0000000000001128 40294087

[B47] Duell P. B. Dutta R. Wolf A. Rosengrant H. (2023). Treatment of cerebrotendinous xanthomatosis in pregnancy: patient and physician perspectives. J. Clin. Lipidol. 17 (5), 700–703. 10.1016/j.jacl.2023.07.002 37543441

[B48] Düfer M. Hörth K. Wagner R. Schittenhelm B. Prowald S. Wagner T. F. J. (2012). Bile acids acutely stimulate insulin secretion of mouse β-cells *via* farnesoid X receptor activation and K(ATP) channel inhibition. Diabetes 61 (6), 1479–1489. 10.2337/db11-0815 22492528 PMC3357280

[B49] Dyson J. K. Beuers U. Jones D. E. J. Lohse A. W. Hudson M. (2018). Primary sclerozing cholangitis. Lancet 391 (10139), 2547–2559. 10.1016/S0140-6736(18)30300-3 29452711

[B50] El-Nabarawi M. Nafady M. Elmenshawe S. Elkarmalawy M. Teaima M. (2021). Liver targeting of daclatasvir *via* tailoring sterically stabilized bilosomes: fabrication, comparative in Vitro/*In Vivo* appraisal and biodistribution studies. Int. J. Nanomedicine 16, 6413–6426. 10.2147/IJN.S319255 34556987 PMC8455511

[B51] Ergenc I. Heneghan M. A. (2025). Elafibranor in the treatment of primary biliary cholangitis. Future Rare Dis. 5 (1), 2580910. 10.1080/23995270.2025.2580910

[B52] Faustino C. Serafim C. Rijo P. Reis C. P. (2016). Bile acids and bile acid derivatives: use in drug delivery systems and as therapeutic agents. Expert Opin. Drug Deliv. 13 (8), 1133–1148. 10.1080/17425247.2016.1178233 27102882

[B53] Ferrell J. M. Chiang J. Y. L. (2021). Bile acid receptors and signaling crosstalk in the liver, gut and brain. Liver Res. 5 (3), 105–118. 10.1016/j.livres.2021.07.002 39957847 PMC11791822

[B54] Fickert P. Hirschfield G. M. Denk G. Marschall H. U. Altorjay I. Färkkilä M. (2017). norUrsodeoxycholic acid improves cholestasis in primary sclerosing cholangitis. J. Hepatology 67 (3), 549–558. 10.1016/j.jhep.2017.05.009 28529147

[B55] Fiorucci S. Distrutti E. (2019a). Chenodeoxycholic acid: an update on its therapeutic applications. Handb. Exp. Pharmacol. 256, 265–282. 10.1007/164_2019_226 31267167

[B56] Fiorucci S. Distrutti E. (2019b). The pharmacology of bile acids and their receptors. Handb. Exp. Pharmacol. 256, 3–18. 10.1007/164_2019_238 31201555

[B57] Fiorucci S. Biagioli M. Sepe V. Zampella A. Distrutti E. (2020). Bile acid modulators for the treatment of nonalcoholic steatohepatitis (NASH). Expert Opin. Investigational Drugs 29 (6), 623–632. 10.1080/13543784.2020.1763302 32552182

[B58] Fiorucci S. Urbani G. Di Giorgio C. Biagioli M. Distrutti E. (2024). Current landscape and evolving therapies for primary biliary cholangitis. Cells 13 (18), 1580. 10.3390/cells13181580 39329760 PMC11429758

[B59] Fleishman J. S. Kumar S. (2024). Bile acid metabolism and signaling in health and disease: molecular mechanisms and therapeutic targets. Signal Transduct. Target. Ther. 9 (1), 97. 10.1038/s41392-024-01811-6 38664391 PMC11045871

[B60] Fligor S. C. Hirsch T. I. Tsikis S. T. Adeola A. Puder M. (2022). Current and emerging adjuvant therapies in biliary atresia. Front. Pediatr. 10, 1007813. 10.3389/fped.2022.1007813 36313875 PMC9614654

[B61] Forrester S. J. Kikuchi D. S. Hernandes M. S. Xu Q. Griendling K. K. (2018). Reactive oxygen species in metabolic and inflammatory signaling. Circulation Res. 122 (6), 877–902. 10.1161/CIRCRESAHA.117.311401 29700084 PMC5926825

[B62] Fuchs C. D. Simbrunner B. Baumgartner M. Campbell C. Reiberger T. Trauner M. (2025). Bile acid metabolism and signalling in liver disease. J. Hepatology 82 (1), 134–153. 10.1016/j.jhep.2024.09.032 39349254

[B63] Gao C. Jiang J. Tan Y. Chen S. (2023). Microglia in neurodegenerative diseases: mechanism and potential therapeutic targets. Signal Transduct. Target. Ther. 8 (1), 359. 10.1038/s41392-023-01588-0 37735487 PMC10514343

[B64] Garcia A. Hsu E. Lin H. C. (2023). Resolution of pruritus in a child with alagille syndrome treated with maralixibat for seven years: durable response and discontinuation of other medications. JPGN Reports 4 (3), e335. 10.1097/PG9.0000000000000335 37600618 PMC10435040

[B65] Graham S. F. Rey N. L. Ugur Z. Yilmaz A. Sherman E. Maddens M. (2018). Metabolomic profiling of bile acids in an experimental model of prodromal parkinson’s disease. Metabolites 8 (4), 71. 10.3390/metabo8040071 30384419 PMC6316593

[B66] Gui W. Hole M. J. Molinaro A. Edlund K. Jørgensen K. K. Su H. (2023). Colitis ameliorates cholestatic liver disease *via* suppression of bile acid synthesis. Nat. Commun. 14 (1), 3304. 10.1038/s41467-023-38840-8 37280200 PMC10244448

[B67] Gunaydin M. Bozkurter Cil A. T. (2018). Progressive familial intrahepatic cholestasis: diagnosis, management, and treatment. Hepatic Med. Evid. Res. 10, 95–104. 10.2147/HMER.S137209 30237746 PMC6136920

[B68] Guo C. Chen W.-D. Wang Y.-D. (2016). TGR5, not only a metabolic regulator. Front. Physiology 7, 646. 10.3389/fphys.2016.00646 28082913 PMC5183627

[B69] Han S. Chiang J. Y. L. (2009). Mechanism of vitamin D receptor inhibition of cholesterol 7alpha-hydroxylase gene transcription in human hepatocytes. Drug Metabolism Dispos. Biol. Fate Chem. 37 (3), 469–478. 10.1124/dmd.108.025155 19106115 PMC2680517

[B70] Hansen M. Sonne D. P. Mikkelsen K. H. Gluud L. L. Vilsbøll T. Knop F. K. (2017). Bile acid sequestrants for glycemic control in patients with type 2 diabetes: a systematic review with meta-analysis of randomized controlled trials. J. Diabetes Its Complicat. 31 (5), 918–927. 10.1016/j.jdiacomp.2017.01.011 28238556

[B71] Harrison S. A. Neff G. Guy C. D. Bashir M. R. Paredes A. H. Frias J. P. (2021). Efficacy and safety of aldafermin, an engineered FGF19 analog, in a randomized, double-blind, placebo-controlled trial of patients with nonalcoholic steatohepatitis. Gastroenterology 160 (1), 219–231. 10.1053/j.gastro.2020.08.004 32781086

[B72] Harrison S. A. Bedossa P. Guy C. D. Schattenberg J. M. Loomba R. Taub R. (2024). A phase 3, randomized, controlled trial of resmetirom in NASH with liver fibrosis. N. Engl. J. Med. 390 (6), 497–509. 10.1056/NEJMoa2309000 38324483

[B73] Hartley J. L. Davenport M. Kelly D. A. (2009). Biliary atresia. Lancet 374 (9702), 1704–1713. 10.1016/S0140-6736(09)60946-6 19914515

[B74] Hasegawa S. Yoneda M. Kurita Y. Nogami A. Honda Y. Hosono K. (2021). Cholestatic liver disease: current treatment strategies and new therapeutic agents. Drugs 81 (10), 1181–1192. 10.1007/s40265-021-01545-7 34142342 PMC8282588

[B75] Hassan S. Hertel P. (2022). Overview of progressive familial intrahepatic cholestasis. Clin. Liver Dis. 26 (3), 371–390. 10.1016/j.cld.2022.03.003 35868680

[B76] Hegade V. S. Kendrick S. F. W. Dobbins R. L. Miller S. R. Thompson D. Richards D. (2017). Effect of ileal bile acid transporter inhibitor GSK2330672 on pruritus in primary biliary cholangitis: a double-blind, randomised, placebo-controlled, crossover, phase 2a study. Lancet London, Engl. 389 (10074), 1114–1123. 10.1016/S0140-6736(17)30319-7 28187915

[B77] Heinz N. Vittorio J. (2023). Treatment of cholestasis in infants and young children. Curr. Gastroenterol. Rep. 25 (11), 344–354. 10.1007/s11894-023-00891-8 37651067

[B78] Heuman D. M. (1989). Quantitative estimation of the hydrophilic-hydrophobic balance of mixed bile salt solutions. J. Lipid Res. 30 (5), 719–730. 10.1016/S0022-2275(20)38331-0 2760545

[B79] Hirschfield G. M. Beuers U. Corpechot C. Invernizzi P. Jones D. Marzioni M. (2017). EASL clinical practice guidelines: the diagnosis and management of patients with primary biliary cholangitis. J. Hepatology 67 (1), 145–172. 10.1016/j.jhep.2017.03.022 28427765

[B80] Hirschfield G. M. Chazouillères O. Drenth J. P. Thorburn D. Harrison S. A. Landis C. S. (2019). Effect of NGM282, an FGF19 analogue, in primary sclerosing cholangitis: a multicenter, randomized, double-blind, placebo-controlled phase II trial. J. Hepatology 70 (3), 483–493. 10.1016/j.jhep.2018.10.035 30414864

[B81] Hirschfield G. M. Bowlus C. L. Mayo M. J. Kremer A. E. Vierling J. M. Kowdley K. V. (2024). A phase 3 trial of seladelpar in primary biliary cholangitis. N. Engl. J. Med. 390 (9), 783–794. 10.1056/NEJMoa2312100 38381664

[B82] Hodge R. J. Nunez D. J. (2016). Therapeutic potential of Takeda-G-protein-receptor-5 (TGR5) agonists. Hope or hype? Diabetes, Obes. and Metabolism 18 (5), 439–443. 10.1111/dom.12636 26818602

[B83] Hofmann A. F. (1999). Bile acids: the good, the bad, and the ugly. Physiology 14 (1), 24–29. 10.1152/physiologyonline.1999.14.1.24 11390813

[B84] Hofmann A. F. Hagey L. R. (2008). Bile acids: chemistry, pathochemistry, biology, pathobiology, and therapeutics. Cell. Molecular Life Sciences CMLS 65 (16), 2461–2483. 10.1007/s00018-008-7568-6 18488143 PMC11131813

[B85] Houri I. Hirschfield G. M. (2024). Primary biliary cholangitis: pathophysiology. Clin. Liver Dis. 28 (1), 79–92. 10.1016/j.cld.2023.06.006 37945164

[B86] Houten S. M. Watanabe M. Auwerx J. (2006). Endocrine functions of bile acids. EMBO J. 25 (7), 1419–1425. 10.1038/sj.emboj.7601049 16541101 PMC1440314

[B87] Hu Y. Sun C. Chen Y. Liu Y. D. Fan J. G. (2024). Pipeline of new drug treatment for non-alcoholic fatty liver disease/metabolic dysfunction-associated steatotic liver disease. J. Clin. Transl. Hepatology 12 (9), 802–814. 10.14218/JCTH.2024.00123 39280073 PMC11393841

[B88] Huang F. Pariante C. M. Borsini A. (2022). From dried bear bile to molecular investigation: a systematic review of the effect of bile acids on cell apoptosis, oxidative stress and inflammation in the brain, across pre-clinical models of neurological, neurodegenerative and neuropsychiatric disorders. Brain, Behav. Immun. 99, 132–146. 10.1016/j.bbi.2021.09.021 34601012

[B89] Huang M. Chen H. Wang H. Zhang Y. Li L. Lan Y. (2025). Global burden and risk factors of MASLD: trends from 1990 to 2021 and predictions to 2030. Intern. Emerg. Med. 20 (4), 1013–1024. 10.1007/s11739-025-03895-6 40019669 PMC12130103

[B90] Hurley M. J. Bates R. Macnaughtan J. Schapira A. H. V. (2022). Bile acids and neurological disease. Pharmacol. and Ther. 240, 108311. 10.1016/j.pharmthera.2022.108311 36400238

[B91] Invernizzi P. Carbone M. Jones D. Levy C. Little N. Wiesel P. (2023). Setanaxib, a first-in-class selective NADPH oxidase 1/4 inhibitor for primary biliary cholangitis: a randomized, placebo-controlled, phase 2 trial. Liver Int. 43 (7), 1507–1522. 10.1111/liv.15596 37183520

[B92] Islam M. Hoggard N. Hadjivassiliou M. (2021). Cerebrotendinous xanthomatosis: diversity of presentation and refining treatment with chenodeoxycholic acid. Cerebellum and Ataxias 8 (1), 5. 10.1186/s40673-021-00128-2 33509302 PMC7844909

[B93] Israelsen M. Francque S. Tsochatzis E. A. Krag A. (2024). Steatotic liver disease. Lancet London, Engl. 404 (10464), 1761–1778. 10.1016/S0140-6736(24)01811-7 39488409

[B94] Ito E. Inuki S. Izumi Y. Takahashi M. Dambayashi Y. Ciacchi L. (2024). Sulfated bile acid is a host-derived ligand for MAIT cells. Sci. Immunol. 9 (91), eade6924. 10.1126/sciimmunol.ade6924 38277465 PMC11147531

[B95] Jalal L. Basaria A. A. A. Yokolo H. (2025). FDA approves first targeted treatment for cerebrotendinous xanthomatosis: a perspective on a landmark in rare lipid storage disease therapy. Health Sci. Rep. 8 (12), e71549. 10.1002/hsr2.71549 41324091 PMC12657251

[B96] Ji L. Ma J. Ma Y. Cheng Z. Gan S. Yuan G. (2025). Berberine ursodeoxycholate for the treatment of type 2 diabetes: a randomized clinical trial. JAMA Netw. Open 8 (3), e2462185. 10.1001/jamanetworkopen.2024.62185 40029660 PMC11877176

[B97] Jiang Y. Li H. Song D. Ye P. Xu N. Chen Y. (2021). Comparative evidence for intrahepatic cholestasis of pregnancy treatment with traditional Chinese medicine therapy: a network meta-analysis. Front. Pharmacol. 12, 774884. 10.3389/fphar.2021.774884 34916949 PMC8670235

[B98] Jin W. Zheng M. Chen Y. Xiong H. (2024). Update on the development of TGR5 agonists for human diseases. Eur. J. Med. Chem. 271, 116462. 10.1016/j.ejmech.2024.116462 38691888

[B99] Joseph Naguib M. Moustafa Kamel A. Thabet Negmeldin A. Elshafeey A. H. Elsayed I. (2020). Molecular docking and statistical optimization of taurocholate-stabilized galactose anchored bilosomes for the enhancement of sofosbuvir absorption and hepatic relative targeting efficiency. Drug Deliv. 27 (1), 996–1009. 10.1080/10717544.2020.1787557 32611266 PMC8216436

[B100] Kalecký K. Bottiglieri T. (2023). Targeted metabolomic analysis in Parkinson’s disease brain frontal cortex and putamen with relation to cognitive impairment. Npj Parkinson’s Dis. 9 (1), 84. 10.1038/s41531-023-00531-y 37270646 PMC10239505

[B101] Kamath B. M. Ye W. Goodrich N. P. Loomes K. M. Romero R. Heubi J. E. (2020). Outcomes of childhood cholestasis in alagille syndrome: results of a multicenter observational study. Hepatol. Commun. 4 (3), 387–398. 10.1002/hep4.1468 33313463 PMC7049675

[B102] Karlsen T. H. Folseraas T. Thorburn D. Vesterhus M. (2017). Primary sclerosing cholangitis – a comprehensive review. J. Hepatology 67 (6), 1298–1323. 10.1016/j.jhep.2017.07.022 28802875

[B103] Kasarla S. S. Garikapati V. Kumar Y. Dodoala S. (2022). Interplay of vitamin D and CYP3A4 polymorphisms in endocrine disorders and cancer. Endocrinol. Metabolism 37 (3), 392–407. 10.3803/EnM.2021.1349 35654576 PMC9262690

[B104] Kaye A. J. Rand E. B. Munoz P. S. Spinner N. B. Flake A. W. Kamath B. M. (2010). Effect of Kasai procedure on hepatic outcome in alagille syndrome. J. Pediatr. Gastroenterology Nutr. 51 (3), 319–321. 10.1097/MPG.0b013e3181df5fd8 20601899

[B105] Keam S. J. (2024). Resmetirom: first approval. Drugs 84 (6), 729–735. 10.1007/s40265-024-02045-0 38771485

[B106] Kempinska-Podhorodecka A. Milkiewicz M. Wasik U. Ligocka J. Zawadzki M. Krawczyk M. (2017). Decreased expression of vitamin D receptor affects an immune response in primary biliary cholangitis *via* the VDR-miRNA155-SOCS1 pathway. Int. J. Mol. Sci. 18 (2), 289. 10.3390/ijms18020289 28146070 PMC5343825

[B107] Khalaf K. Tornese P. Cocco A. Albanese A. (2022). Tauroursodeoxycholic acid: a potential therapeutic tool in neurodegenerative diseases. Transl. Neurodegener. 11 (1), 33. 10.1186/s40035-022-00307-z 35659112 PMC9166453

[B108] Khan M. A. B. Hashim M. J. King J. K. Govender R. D. Mustafa H. Al Kaabi J. (2020). Epidemiology of type 2 diabetes – global burden of disease and forecasted trends. J. Epidemiol. Glob. Health 10 (1), 107–111. 10.2991/jegh.k.191028.001 32175717 PMC7310804

[B109] Kim R. Lee J. Y. Park S. Han K. Shin C. M. (2021). Cholecystectomy and subsequent risk of Parkinson’s disease: a nationwide retrospective cohort study. Npj Parkinson’s Dis. 7 (1), 100. 10.1038/s41531-021-00245-z 34785689 PMC8595409

[B110] Kimura M. Ogawa E. Harada K. Imamura J. Saio M. Ikura Y. (2022). Feasibility, safety and tolerability of the CREB-Binding protein/β-catenin inhibitor OP-724 in patients with advanced primary biliary cholangitis: an investigator-initiated, open-label, non-randomised, two-centre, phase 1 study. BMJ Open Gastroenterology 9 (1), e001001. 10.1136/bmjgast-2022-001001 36442892 PMC9710334

[B111] Kimura M. Nishikawa K. Osawa Y. Imamura J. Yamaji K. Harada K. (2022). Inhibition of CBP/β-catenin signaling ameliorated fibrosis in cholestatic liver disease. Hepatol. Commun. 6 (10), 2732–2747. 10.1002/hep4.2043 35855613 PMC9512479

[B112] Kisanuki Y. Y. Nobrega P. R. Himes R. Jayadev S. Bernat J. A. Prakash V. (2025). Efficacy, safety, and tolerability of chenodeoxycholic acid (CDCA) in adult patients with cerebrotendinous xanthomatosis (RESTORE): a randomized withdrawal, double-blind, placebo-controlled, crossover phase-3 study. Genet. Med. 27 (7), 101449. 10.1016/j.gim.2025.101449 40297984

[B113] Kohut T. J. Gilbert M. A. Loomes K. M. (2021). Alagille syndrome: a focused review on clinical features, genetics, and treatment. Seminars Liver Dis. 41 (4), 525–537. 10.1055/s-0041-1730951 34215014

[B114] Kong X. Kong Y. Zhang F. Wang T. Yan J. (2016). Evaluating the effectiveness and safety of ursodeoxycholic acid in treatment of intrahepatic cholestasis of pregnancy. Medicine 95 (40), e4949. 10.1097/MD.0000000000004949 27749550 PMC5059052

[B115] Kotb M. A. (2008). Review of historical cohort: ursodeoxycholic acid in extrahepatic biliary atresia. J. Pediatr. Surg. 43 (7), 1321–1327. 10.1016/j.jpedsurg.2007.11.043 18639689

[B116] Kothari S. Afshar Y. Friedman L. S. Ahn J. (2024). AGA clinical practice update on pregnancy-related gastrointestinal and liver disease: expert review. Gastroenterology 167 (5), 1033–1045. 10.1053/j.gastro.2024.06.014 39140906

[B117] Kowdley K. V. Vuppalanchi R. Levy C. Floreani A. Andreone P. LaRusso N. F. (2020). A randomized, placebo-controlled, phase II study of obeticholic acid for primary sclerosing cholangitis. J. Hepatology 73 (1), 94–101. 10.1016/j.jhep.2020.02.033 32165251 PMC8157171

[B118] Kowdley K. V. Bowlus C. L. Levy C. Akarca U. S. Alvares-da-Silva M. R. Andreone P. (2024). Efficacy and safety of elafibranor in primary biliary cholangitis. N. Engl. J. Med. 390 (9), 795–805. 10.1056/NEJMoa2306185 37962077

[B119] Kremer A. E. Beuers U. Oude-Elferink R. P. J. Pusl T. (2008). Pathogenesis and treatment of pruritus in cholestasis. Drugs 68 (15), 2163–2182. 10.2165/00003495-200868150-00006 18840005

[B120] Kuang J. Wang J. Li Y. Li M. Zhao M. Ge K. (2023). Hyodeoxycholic acid alleviates non-alcoholic fatty liver disease through modulating the gut-liver axis. Cell Metab. 35 (10), 1752–1766.e8. 10.1016/j.cmet.2023.07.011 37591244

[B121] Kubitz R. Dröge C. Stindt J. Weissenberger K. Häussinger D. (2012). The bile salt export pump (BSEP) in health and disease. Clin. Res. Hepatology Gastroenterology 36 (6), 536–553. 10.1016/j.clinre.2012.06.006 22795478

[B122] Kuhre R. E. Wewer Albrechtsen N. J. Larsen O. Jepsen S. L. Balk-Møller E. Andersen D. B. (2018). Bile acids are important direct and indirect regulators of the secretion of appetite- and metabolism-regulating hormones from the gut and pancreas. Mol. Metab. 11, 84–95. 10.1016/j.molmet.2018.03.007 29656109 PMC6001409

[B123] Lakić B. Škrbić R. Uletilović S. Mandić-Kovačević N. Grabež M. Šarić M. P. (2024). Beneficial effects of ursodeoxycholic acid on metabolic parameters and oxidative stress in patients with type 2 diabetes mellitus: a randomized double-blind, placebo-controlled clinical study. J. Diabetes Res. 2024, 4187796. 10.1155/2024/4187796 38455850 PMC10919985

[B124] Lee Y. Y. Hong S. H. Lee Y. J. Chung S. S. Jung H. S. Park S. G. (2010). Tauroursodeoxycholate (TUDCA), chemical chaperone, enhances function of islets by reducing ER stress. Biochem. Biophysical Res. Commun. 397 (4), 735–739. 10.1016/j.bbrc.2010.06.022 20541525

[B125] Lee R. H. Lee R. H. Mara Greenberg M. Metz T. D. Pettker C. M. (2021). Society for maternal-fetal medicine consult series #53: intrahepatic cholestasis of pregnancy: replaces consult #13, April 2011. Am. J. Obstetrics and Gynecol. 224 (2), B2–B9. 10.1016/j.ajog.2020.11.002 33197417

[B126] Leibovitzh H. Nayeri S. Borowski K. Hernandez-Rocha C. Lee S. H. Turpin W. (2024). Inflammatory bowel disease associated with primary sclerosing cholangitis is associated with an altered gut microbiome and bile acid profile. J. Crohn’s Colitis 18 (12), 1957–1966. 10.1093/ecco-jcc/jjae096 38980940 PMC11637524

[B127] Lenci I. Milana M. Signorello A. Grassi G. Baiocchi L. (2023). Secondary bile acids and the biliary epithelia: the good and the bad. World J. Gastroenterology 29 (2), 357–366. 10.3748/wjg.v29.i2.357 36687129 PMC9846939

[B128] Levy C. Bowlus C. L. (2025). Primary biliary cholangitis: personalizing second-line therapies. Hepatol. Baltim. Md 82 (4), 895–910. 10.1097/HEP.0000000000001166 39707635

[B129] Levy C. Manns M. Hirschfield G. (2023). New treatment paradigms in primary biliary cholangitis. Clin. Gastroenterology Hepatology Official Clin. Pract. J. Am. Gastroenterological Assoc. 21 (8), 2076–2087. 10.1016/j.cgh.2023.02.005 36809835

[B130] Levy C. Buchanan-Peart K.-A. MacEwan J. P. Levine A. Nair R. Wheeler D. (2025a). A nationwide study of primary biliary cholangitis prevalence, geographic distribution, and health care providers. Hepatol. Commun. 9 (5), e0677. 10.1097/HC9.0000000000000677 40227093 PMC11999412

[B131] Levy C. Abouda G. F. Bilir B. M. Bonder A. Bowlus C. L. Campos-Varela I. (2025b). Safety and efficacy of elafibranor in primary sclerosing cholangitis: the ELMWOOD phase II randomized-controlled trial. J. Hepatology 84 (25), S0168–S8278. 10.1016/j.jhep.2025.04.025 40350321

[B132] Lewis J. D. Parlett L. E. Jonsson Funk M. L. Brensinger C. Pate V. Wu Q. (2023). Incidence, prevalence, and racial and ethnic distribution of inflammatory bowel disease in the United States. Gastroenterology 165 (5), 1197–1205.e2. 10.1053/j.gastro.2023.07.003 37481117 PMC10592313

[B133] Li T. Apte U. (2015). “Bile acid metabolism and signaling in cholestasis, inflammation, and cancer,” in Advances in pharmacology (Elsevier), 263–302. 10.1016/bs.apha.2015.04.003 PMC4615692 26233910

[B134] Li T. Chiang J. Y. L. (2014). Bile acid signaling in metabolic disease and drug therapy. Pharmacol. Rev. 66 (4), 948–983. 10.1124/pr.113.008201 25073467 PMC4180336

[B135] Li T. Francl J. M. Boehme S. Ochoa A. Zhang Y. Klaassen C. D. (2012). Glucose and insulin induction of bile acid synthesis: mechanisms and implication in diabetes and obesity. J. Biol. Chem. 287 (3), 1861–1873. 10.1074/jbc.M111.305789 22144677 PMC3265867

[B136] Li P. Killinger B. A. Ensink E. Beddows I. Yilmaz A. Lubben N. (2021). Gut microbiota dysbiosis is associated with elevated bile acids in parkinson’s disease. Metabolites 11 (1), 29. 10.3390/metabo11010029 33406628 PMC7823437

[B137] Lin X. Mai M. He T. Huang H. Zhang P. Xia E. (2022). Efficiency of ursodeoxycholic acid for the treatment of nonalcoholic steatohepatitis: a systematic review and meta-analysis. Expert Rev. Gastroenterology and Hepatology 16 (6), 537–545. 10.1080/17474124.2022.2083605 35617696

[B138] Lin J. Nie Q. Cheng J. Zhong Y. N. Zhang T. Zhang X. (2025). A microbial amino-acid-conjugated bile acid, tryptophan-cholic acid, improves glucose homeostasis *via* the orphan receptor MRGPRE. Cell 0 (0), 4530–4548.e25. 10.1016/j.cell.2025.05.010 40446798

[B139] Lingappan K. (2018). NF-κB in oxidative stress. Curr. Opin. Toxicol. 7, 81–86. 10.1016/j.cotox.2017.11.002 29862377 PMC5978768

[B140] Liu J. Lu H. Lu Y. F. Lei X. Cui J. Y. Ellis E. (2014). Potency of individual bile acids to regulate bile acid synthesis and transport genes in primary human hepatocyte cultures. Toxicol. Sci. An Official J. Soc. Toxicol. 141 (2), 538–546. 10.1093/toxsci/kfu151 25055961 PMC4271050

[B141] Liu T. Zhang L. Joo D. Sun S. C. (2017). NF-κB signaling in inflammation. Signal Transduct. Target. Ther. 2 (1), 17023. 10.1038/sigtrans.2017.23 29158945 PMC5661633

[B142] Liu C. Li M. K. Alster T. S. (2021). Alternative cosmetic and medical applications of injectable deoxycholic acid: a systematic review. Dermatol. Surg. 47 (11), 1466–1472. 10.1097/DSS.0000000000003159 34537786

[B143] Loh J. S. Mak W. Q. Tan L. K. S. Ng C. X. Chan H. H. Yeow S. H. (2024). Microbiota–gut–brain axis and its therapeutic applications in neurodegenerative diseases. Signal Transduct. Target. Ther. 9 (1), 37. 10.1038/s41392-024-01743-1 38360862 PMC10869798

[B144] Ludwig D. R. Anderson M. A. Itani M. Sharbidre K. G. Lalwani N. Paspulati R. M. (2023). Secondary sclerosing cholangitis: mimics of primary sclerosing cholangitis. Abdom. Radiol. (New York) 48 (1), 151–165. 10.1007/s00261-022-03551-z 35585354 PMC9116710

[B145] MahmoudianDehkordi S. Arnold M. Nho K. Ahmad S. Jia W. Xie G. (2019). Altered bile acid profile associates with cognitive impairment in alzheimer’s disease – an emerging role for gut microbiome. Alzheimer’s and Dementia The Journal Alzheimer’s Assoc. 15 (1), 76–92. 10.1016/j.jalz.2018.07.217 30337151 PMC6487485

[B146] Makishima M. Lu T. T. Xie W. Whitfield G. K. Domoto H. Evans R. M. (2002). Vitamin D receptor as an intestinal bile acid sensor. Science 296 (5571), 1313–1316. 10.1126/science.1070477 12016314

[B147] Mandia D. Chaussenot A. Besson G. Lamari F. Castelnovo G. Curot J. (2019). Cholic acid as a treatment for cerebrotendinous xanthomatosis in adults. J. Neurology 266 (8), 2043–2050. 10.1007/s00415-019-09377-y 31115677

[B148] Manns M. P. Bergquist A. Karlsen T. H. Levy C. Muir A. J. Ponsioen C. (2025). Primary sclerosing cholangitis. Nat. Rev. Dis. Prim. 11 (1), 17. 10.1038/s41572-025-00600-x 40082445

[B149] Mao Z. Hui H. Zhao X. Xu L. Qi Y. Yin L. (2023). Protective effects of dioscin against Parkinson’s disease *via* regulating bile acid metabolism through remodeling gut microbiome/GLP-1 signaling. J. Pharm. Analysis 13 (10), 1153–1167. 10.1016/j.jpha.2023.06.007 38024855 PMC10657977

[B150] Masubuchi N. Sugihara M. Sugita T. Amano K. Nakano M. Matsuura T. (2016). Oxidative stress markers, secondary bile acids and sulfated bile acids classify the clinical liver injury type: promising diagnostic biomarkers for cholestasis. Chemico-Biological Interact. 255, 83–91. 10.1016/j.cbi.2015.08.016 26325587

[B151] Matloub A. A. Salama A. H. Aglan H. A. AbouSamra M. M. ElSouda S. S. M. Ahmed H. H. (2018). Exploiting bilosomes for delivering bioactive polysaccharide isolated from Enteromorpha intestinalis for hacking hepatocellular carcinoma. Drug Dev. Industrial Pharm. 44 (4), 523–534. 10.1080/03639045.2017.1402922 29115890

[B152] McArthur K. Kile B. T. (2018). Apoptotic caspases: multiple or mistaken identities? Trends Cell Biol. 28 (6), 475–493. 10.1016/j.tcb.2018.02.003 29551258

[B153] McKiernan P. Bernabeu J. Q. Girard M. Indolfi G. Lurz E. Trivedi P. (2023). Opinion paper on the diagnosis and treatment of progressive familial intrahepatic cholestasis. JHEP Rep. 6 (1), 100949. 10.1016/j.jhepr.2023.100949 38192535 PMC10772241

[B154] Meixiong J. Vasavda C. Snyder S. H. Dong X. (2019). MRGPRX4 is a G protein-coupled receptor activated by bile acids that May contribute to cholestatic pruritus. Proc. Natl. Acad. Sci. U. S. A. 116 (21), 10525–10530. 10.1073/pnas.1903316116 31068464 PMC6535009

[B155] Meixiong J. Vasavda C. Green D. Zheng Q. Qi L. Kwatra S. G. (2025). Identification of a bilirubin receptor that May mediate a component of cholestatic itch. eLife 8, e44116. 10.7554/eLife.44116 30657454 PMC6368403

[B156] Miethke A. G. Moukarzel A. Porta G. Covarrubias Esquer J. Czubkowski P. Ordonez F. (2024). Maralixibat in progressive familial intrahepatic cholestasis (MARCH-PFIC): a multicentre, randomised, double-blind, placebo-controlled, phase 3 trial. Lancet. Gastroenterology and Hepatology 9 (7), 620–631. 10.1016/S2468-1253(24)00080-3 38723644 PMC13409310

[B157] Mighiu C. O'Hara S. Ferri Grazzi E. Murray K. F. Schattenberg J. M. Ventura E. (2022). Impact of progressive familial intrahepatic cholestasis on caregivers: caregiver-reported outcomes from the multinational PICTURE study. Orphanet J. Rare Dis. 17 (1), 32. 10.1186/s13023-022-02177-0 35109890 PMC8809495

[B158] Mitchell E. Gilbert M. Loomes K. M. (2018). Alagille syndrome. Clin. Liver Dis. 22 (4), 625–641. 10.1016/j.cld.2018.06.001 30266153

[B159] Mitrović D. Zaklan D. Đanić M. Stanimirov B. Stankov K. Al-Salami H. (2025). The pharmaceutical and pharmacological potential applications of bilosomes as nanocarriers for drug delivery. Molecules 30 (5), 1181. 10.3390/molecules30051181 40076403 PMC11901966

[B160] Mudaliar S. Henry R. R. Sanyal A. J. Morrow L. Marschall H. U. Kipnes M. (2013). Efficacy and safety of the farnesoid X receptor agonist obeticholic acid in patients with type 2 diabetes and nonalcoholic fatty liver disease. Gastroenterology 145(3) 574–582e1. 10.1053/j.gastro.2013.05.042 23727264

[B161] Muir A. J. Levy C. Janssen H. L. A. Montano-Loza A. J. Shiffman M. L. Caldwell S. (2019). Simtuzumab for primary sclerosing cholangitis: phase 2 study results with insights on the natural history of the disease. Hepatology 69 (2), 684–698. 10.1002/hep.30237 30153359

[B162] Mulak A. (2021). Bile acids as key modulators of the brain-gut-microbiota axis in alzheimer’s disease. J. Alzheimer’s Disease JAD 84 (2), 461–477. 10.3233/JAD-210608 34569953 PMC8673511

[B163] Nauck M. A. Wefers J. Meier J. J. (2021). Treatment of type 2 diabetes: challenges, hopes, and anticipated successes. Lancet Diabetes and Endocrinol. 9 (8), 525–544. 10.1016/S2213-8587(21)00113-3 34181914

[B164] Nayak D. Rathnanand M. Tippavajhala V. K. (2023). Unlocking the potential of bilosomes and modified bilosomes: a comprehensive journey into advanced drug delivery trends. AAPS PharmSciTech 24 (8), 238. 10.1208/s12249-023-02696-4 37989979

[B165] Neuschwander-Tetri B. A. Loomba R. Sanyal A. J. Lavine J. E. Van Natta M. L. Abdelmalek M. F. (2015). Farnesoid X nuclear receptor ligand obeticholic acid for non-cirrhotic, non-alcoholic steatohepatitis (FLINT): a multicentre, randomised, placebo-controlled trial. Lancet London, Engl. 385 (9972), 956–965. 10.1016/S0140-6736(14)61933-4 25468160 PMC4447192

[B166] Nevens F. Andreone P. Mazzella G. Strasser S. I. Bowlus C. Invernizzi P. (2016). A placebo-controlled trial of obeticholic acid in primary biliary cholangitis. N. Engl. J. Med. 375 (7), 631–643. 10.1056/NEJMoa1509840 27532829

[B167] Newsome P. N. Palmer M. Freilich B. Sheikh M. Y. Sheikh A. Sarles H. (2020). Volixibat in adults with non-alcoholic steatohepatitis: 24-Week interim analysis from a randomized, phase II study. J. Hepatology 73 (2), 231–240. 10.1016/j.jhep.2020.03.024 32234329

[B168] Nóbrega P. R. Bernardes A. M. Ribeiro R. M. Vasconcelos S. C. Araújo D. A. B. S. Gama V. C. d. V. (2022). Cerebrotendinous xanthomatosis: a practice review of pathophysiology, diagnosis, and treatment. Front. Neurology 13, 1049850. 10.3389/fneur.2022.1049850 36619921 PMC9816572

[B169] Nowell J. Blunt E. Edison P. (2023). Incretin and insulin signaling as novel therapeutic targets for Alzheimer’s and Parkinson’s disease. Mol. Psychiatry 28 (1), 217–229. 10.1038/s41380-022-01792-4 36258018 PMC9812772

[B170] Ovadia C. Sajous J. Seed P. T. Patel K. Williamson N. J. Attilakos G. (2021). Ursodeoxycholic acid in intrahepatic cholestasis of pregnancy: a systematic review and individual participant data meta-analysis. Lancet. Gastroenterology and Hepatology 6 (7), 547–558. 10.1016/S2468-1253(21)00074-1 33915090 PMC8192305

[B171] Ovchinsky N. Aumar M. Baker A. Baumann U. Bufler P. Cananzi M. (2024). Efficacy and safety of odevixibat in patients with alagille syndrome (ASSERT): a phase 3, double-blind, randomised, placebo-controlled trial. Lancet. Gastroenterology and Hepatology 9 (7), 632–645. 10.1016/S2468-1253(24)00074-8 38670135

[B172] Pan X. Elliott C. T. McGuinness B. Passmore P. Kehoe P. G. Hölscher C. (2017). Metabolomic profiling of bile acids in clinical and experimental samples of alzheimer’s disease. Metabolites 7 (2), 28. 10.3390/metabo7020028 28629125 PMC5487999

[B173] Pan Y. Zhang H. Li M. He T. Guo S. Zhu L. (2024). Novel approaches in IBD therapy: targeting the gut microbiota-bile acid axis. Gut Microbes 16 (1), 2356284. 10.1080/19490976.2024.2356284 38769683 PMC11110704

[B174] Pataia V. Dixon P. H. Williamson C. (2017). Pregnancy and bile acid disorders. Am. J. Physiology. Gastrointest. Liver Physiology 313 (1), G1–G6. 10.1152/ajpgi.00028.2017 28450276

[B175] Patel S. P. Vasavda C. Ho B. Meixiong J. Dong X. Kwatra S. G. (2019). Cholestatic pruritus: emerging mechanisms and therapeutics. J. Am. Acad. Dermatology 81 (6), 1371–1378. 10.1016/j.jaad.2019.04.035 31009666 PMC7825249

[B176] Patel K. Harrison S. A. Elkhashab M. Trotter J. F. Herring R. Rojter S. E. (2020). Cilofexor, a nonsteroidal FXR agonist, in patients with noncirrhotic NASH: a phase 2 randomized controlled trial. Hepatol. Baltim. Md 72 (1), 58–71. 10.1002/hep.31205 32115759

[B177] Paumgartner G. Beuers U. (2002). Ursodeoxycholic acid in cholestatic liver disease: mechanisms of action and therapeutic use revisited. Hepatol. Baltim. Md 36 (3), 525–531. 10.1053/jhep.2002.36088 12198643

[B178] Pavek P. Pospechova K. Svecova L. Syrova Z. Stejskalova L. Blazkova J. (2010). Intestinal cell-specific vitamin D receptor (VDR)-Mediated transcriptional regulation of CYP3A4 gene. Biochem. Pharmacol. 79 (2), 277–287. 10.1016/j.bcp.2009.08.017 19712670

[B179] Payne T. Appleby M. Buckley E. van Gelder L. M. A. Mullish B. H. Sassani M. (2023). A double-blind, randomized, placebo-controlled trial of ursodeoxycholic acid (UDCA) in parkinson’s disease. Mov. Disord. 38 (8), 1493–1502. 10.1002/mds.29450 37246815 PMC10527073

[B180] Perez M. J. Briz O. (2009). Bile-acid-induced cell injury and protection. World J. Gastroenterology. WJG 15 (14), 1677–1689. 10.3748/wjg.15.1677 19360911 PMC2668773

[B181] Pols T. W. H. Noriega L. G. Nomura M. Auwerx J. Schoonjans K. (2011). The bile acid membrane receptor TGR5 as an emerging target in metabolism and inflammation. J. Hepatology 54 (6), 1263–1272. 10.1016/j.jhep.2010.12.004 21145931 PMC3650458

[B182] Prikhodko V. A. Bezborodkina N. N. Okovityi S. V. (2022). Pharmacotherapy for non-alcoholic fatty liver disease: emerging targets and drug candidates. Biomedicines 10 (2), 274. 10.3390/biomedicines10020274 35203484 PMC8869100

[B183] Priya S. Desai V. M. Singhvi G. (2023). Surface modification of lipid-based nanocarriers: a potential approach to enhance targeted drug delivery. ACS Omega 8 (1), 74–86. 10.1021/acsomega.2c05976 36643539 PMC9835629

[B184] Pusl T. Beuers U. (2006). Ursodeoxycholic acid treatment of vanishing bile duct syndromes. World J. Gastroenterology. WJG 12 (22), 3487–3495. 10.3748/wjg.v12.i22.3487 16773706 PMC4087565

[B185] Qiu J.-L. Shao M. Y. Xie W. F. Li Y. Yang H. D. Niu M. M. (2018). Effect of combined ursodeoxycholic acid and glucocorticoid on the outcome of Kasai procedure: a systematic review and meta-analysis. Medicine 97 (35), e12005. 10.1097/MD.0000000000012005 30170405 PMC6393119

[B186] Quinn M. McMillin M. Galindo C. Frampton G. Pae H. Y. DeMorrow S. (2014). Bile acids permeabilize the blood brain barrier after bile duct ligation. Dig. Liver Disease. Official Journal Italian Soc. Gastroenterology Italian Assoc. Study Liver 46 (6), 527–534. 10.1016/j.dld.2014.01.159 24629820 PMC4065628

[B187] Quinn R. A. Melnik A. V. Vrbanac A. Fu T. Patras K. A. Christy M. P. (2020). Global chemical effects of the microbiome include new bile-acid conjugations. Nature 579 (7797), 123–129. 10.1038/s41586-020-2047-9 32103176 PMC7252668

[B188] Rajbhar N. Singhal D. Nijhawan H. P. Verma P. Soni G. Yadav K. S. (2025). Bilosomes as a novel ocular drug delivery system: assessing the material attributes, process parameters, and quality attributes. Exp. Eye Res. 255, 110364. 10.1016/j.exer.2025.110364 40157630

[B189] Ratziu V. de Guevara L. Safadi R. Poordad F. Fuster F. Flores-Figueroa J. (2021). Aramchol in patients with nonalcoholic steatohepatitis: a randomized, double-blind, placebo-controlled phase 2b trial. Nat. Medicine 27 (10), 1825–1835. 10.1038/s41591-021-01495-3 34621052 PMC12165723

[B190] Ratziu V. Rinella M. E. Neuschwander-Tetri B. A. Lawitz E. Denham D. Kayali Z. (2022). EDP-305 in patients with NASH: a phase II double-blind placebo-controlled dose-ranging study. J. Hepatology 76 (3), 506–517. 10.1016/j.jhep.2021.10.018 34740705

[B191] Ratziu V. Yilmaz Y. Lazas D. Friedman S. L. Lackner C. Behling C. (2025). Aramchol improves hepatic fibrosis in metabolic dysfunction-associated steatohepatitis: results of multimodality assessment using both conventional and digital pathology. Hepatol. Baltim. Md 81 (3), 932–946. 10.1097/HEP.0000000000000980 38916482 PMC12186543

[B192] Reich M. Deutschmann K. Sommerfeld A. Klindt C. Kluge S. Kubitz R. (2016). TGR5 is essential for bile acid-dependent cholangiocyte proliferation *in vivo* and *in vitro* . Gut 65 (3), 487–501. 10.1136/gutjnl-2015-309458 26420419

[B193] Rinella M. E. Lieu H. D. Kowdley K. V. Goodman Z. D. Alkhouri N. Lawitz E. (2024). A randomized, double-blind, placebo-controlled trial of aldafermin in patients with NASH and compensated cirrhosis. Hepatol. Baltim. Md 79 (3), 674–689. 10.1097/HEP.0000000000000607 37732990 PMC10871650

[B194] Sadeghi A. (2024). Global incidence of intrahepatic cholestasis of pregnancy: a protocol for systematic review and meta-analysis. Health Sci. Rep. 7 (2), e1901. 10.1002/hsr2.1901 38361799 PMC10867694

[B195] Safadi R. Konikoff F. M. Mahamid M. Zelber-Sagi S. Halpern M. Gilat T. (2014). “The fatty acid-bile acid conjugate aramchol reduces liver fat content in patients with nonalcoholic fatty liver disease,” Clin. Gastroenterology Hepatology Official Clin. Pract. J. Am. Gastroenterological Assoc. 12(12) 2085–2091e1. 10.1016/j.cgh.2014.04.038 24815326

[B196] Salic K. Kleemann R. Wilkins-Port C. McNulty J. Verschuren L. Palmer M. (2019). Apical sodium-dependent bile acid transporter inhibition with volixibat improves metabolic aspects and components of non-alcoholic steatohepatitis in Ldlr-/-.Leiden mice. PloS One 14 (6), e0218459. 10.1371/journal.pone.0218459 31233523 PMC6590809

[B197] Sampson T. R. Debelius J. W. Thron T. Janssen S. Shastri G. G. Ilhan Z. E. (2016). Gut microbiota regulate motor deficits and neuroinflammation in a model of parkinson’s disease. Cell 167 (6), 1469–1480.e12. 10.1016/j.cell.2016.11.018 27912057 PMC5718049

[B198] Sanyal A. J. Lopez P. Lawitz E. J. Lucas K. J. Loeffler J. Kim W. (2023). Tropifexor for nonalcoholic steatohepatitis: an adaptive, randomized, placebo-controlled phase 2a/b trial. Nat. Med. 29 (2), 392–400. 10.1038/s41591-022-02200-8 36797481 PMC9941046

[B199] Sarcognato S. Sacchi D. Grillo F. Cazzagon N. Fabris L. Cadamuro M. (2021). Autoimmune biliary diseases: primary biliary cholangitis and primary sclerosing cholangitis. Pathologica 113 (3), 170–184. 10.32074/1591-951X-245 34294935 PMC8299325

[B200] Šarenac T. M. Mikov M. (2018). Bile acid synthesis: from nature to the chemical modification and synthesis and their applications as drugs and nutrients. Front. Pharmacol. 9, 939. 10.3389/fphar.2018.00939 30319399 PMC6168039

[B201] Sasaki H. Masuno H. Kawasaki H. Yoshihara A. Numoto N. Ito N. (2021). Lithocholic acid derivatives as potent vitamin D receptor agonists. J. Med. Chem. 64 (1), 516–526. 10.1021/acs.jmedchem.0c01420 33369416

[B202] Schwarz M. (2004). Pathways and defects of bile acid synthesis: insights from *in vitro* and *in vivo* experimental models. Drug Discov. Today Dis. Models 1 (3), 205–212. 10.1016/j.ddmod.2004.10.004

[B203] Shamsi A. Anwar S. Mohammad T. Alajmi M. F. Hussain A. Rehman M. T. (2020). MARK4 inhibited by AChE inhibitors, donepezil and rivastigmine tartrate: insights into alzheimer’s disease therapy. Biomolecules 10 (5), 789. 10.3390/biom10050789 32443670 PMC7277793

[B204] Shao Y. Li T. Liu Z. Wang X. Xu X. Li S. (2021). Comprehensive metabolic profiling of Parkinson’s disease by liquid chromatography-mass spectrometry. Mol. Neurodegener. 16 (1), 4. 10.1186/s13024-021-00425-8 33485385 PMC7825156

[B205] Shima K. R. Ota T. Kato K. I. Takeshita Y. Misu H. Kaneko S. (2018). Ursodeoxycholic acid potentiates dipeptidyl peptidase-4 inhibitor sitagliptin by enhancing glucagon-like peptide-1 secretion in patients with type 2 diabetes and chronic liver disease: a pilot randomized controlled and add-on study. BMJ Open Diabetes Research and Care 6 (1), e000469. 10.1136/bmjdrc-2017-000469 29607050 PMC5873545

[B206] Shukla A. Mishra V. Kesharwani P. (2016). Bilosomes in the context of oral immunization: development, challenges and opportunities. Drug Discov. Today 21 (6), 888–899. 10.1016/j.drudis.2016.03.013 27038539

[B207] Shulpekova Y. Shirokova E. Zharkova M. Tkachenko P. Tikhonov I. Stepanov A. (2022). A recent ten-year perspective: bile acid metabolism and signaling. Molecules 27 (6), 1983. 10.3390/molecules27061983 35335345 PMC8953976

[B208] Simbrunner B. Paternostro R. Reiberger T. Trauner M. (2025). Bile acid signaling in MASLD: from pathogenesis to therapeutic applications. Hepatol. Baltim. Md. [Preprint]. 10.1097/HEP.0000000000001539 40971685

[B209] Smith D. D. Rood K. M. (2020). Intrahepatic cholestasis of pregnancy. Clin. Obstetrics Gynecol. 63 (1), 134–151. 10.1097/GRF.0000000000000495 31764000

[B210] Sonne D. P. Hansen M. Knop F. K. (2014). Bile acid sequestrants in type 2 diabetes: potential effects on GLP1 secretion. Eur. J. Endocrinol. 171 (2), R47–R65. 10.1530/EJE-14-0154 24760535

[B211] Stapelbroek J. M. van Erpecum K. J. Klomp L. W. J. Houwen R. H. J. (2010). Liver disease associated with canalicular transport defects: current and future therapies. J. Hepatology 52 (2), 258–271. 10.1016/j.jhep.2009.11.012 20034695

[B212] Steinberg G. R. Carpentier A. C. Wang D. (2025). MASH: the nexus of metabolism, inflammation, and fibrosis. J. Clin. Investigation 135 (18), e186420. 10.1172/JCI186420 40955659 PMC12435842

[B213] Sun J. Li H. Jin Y. Yu J. Mao S. Su K. P. (2021). Probiotic *Clostridium butyricum* ameliorated motor deficits in a mouse model of Parkinson’s disease *via* gut microbiota-GLP-1 pathway. Brain, Behav. Immun. 91, 703–715. 10.1016/j.bbi.2020.10.014 33148438

[B214] Sun X. Shukla M. Wang W. Li S. (2024). Unlocking gut-liver-brain axis communication metabolites: energy metabolism, immunity and barriers. Npj Biofilms Microbiomes 10 (1), 136. 10.1038/s41522-024-00610-9 39587086 PMC11589602

[B215] Sutton H. Karpen S. J. Kamath B. M. (2024). Pediatric cholestatic diseases: common and unique pathogenic mechanisms. Annu. Rev. Pathology 19, 319–344. 10.1146/annurev-pathmechdis-031521-025623 38265882

[B216] Takahashi S. Luo Y. Ranjit S. Xie C. Libby A. E. Orlicky D. J. (2020). Bile acid sequestration reverses liver injury and prevents progression of nonalcoholic steatohepatitis in Western diet–fed mice. J. Biol. Chem. 295 (14), 4733–4747. 10.1074/jbc.RA119.011913 32075905 PMC7135973

[B217] Tan X. Xiang Y. Shi J. Chen L. Yu D. (2024). Targeting NTCP for liver disease treatment: a promising strategy. J. Pharm. Analysis 14 (9), 100979. 10.1016/j.jpha.2024.100979 39310850 PMC11415714

[B218] Tanaka A. Ma X. Takahashi A. Vierling J. M. (2024). Primary biliary cholangitis. Lancet 404 (10457), 1053–1066. 10.1016/S0140-6736(24)01303-5 39216494

[B219] Tang B. Tang L. Li S. Liu S. He J. Li P. (2023). Gut microbiota alters host bile acid metabolism to contribute to intrahepatic cholestasis of pregnancy. Nat. Commun. 14, 1305. 10.1038/s41467-023-36981-4 36894566 PMC9998625

[B220] Tang M. Xiong L. Cai J. Fu J. Liu H. Ye Y. (2024). Intrahepatic cholestasis of pregnancy: insights into pathogenesis and advances in omics studies. Hepatol. Int. 18 (1), 50–62. 10.1007/s12072-023-10604-y 37957532

[B221] Targher G. Valenti L. Byrne C. D. (2025). Metabolic dysfunction-associated steatotic liver disease. N. Engl. J. Med. 393 (7), 683–698. 10.1056/NEJMra2412865 40802944

[B222] Thomas C. Pellicciari R. Pruzanski M. Auwerx J. Schoonjans K. (2008). Targeting bile-acid signalling for metabolic diseases. Nat. Rev. Drug Discov. 7 (8), 678–693. 10.1038/nrd2619 18670431

[B223] Thomas J. P. Modos D. Rushbrook S. M. Powell N. Korcsmaros T. (2022). The emerging role of bile acids in the pathogenesis of inflammatory bowel disease. Front. Immunol. 13, 829525. 10.3389/fimmu.2022.829525 35185922 PMC8850271

[B224] Thompson R. J. Arnell H. Artan R. Baumann U. Calvo P. L. Czubkowski P. (2022). Odevixibat treatment in progressive familial intrahepatic cholestasis: a randomised, placebo-controlled, phase 3 trial. Lancet. Gastroenterology and Hepatology 7 (9), 830–842. 10.1016/S2468-1253(22)00093-0 35780807

[B225] Trauner M. Gulamhusein A. Hameed B. Caldwell S. Shiffman M. L. Landis C. (2019). The nonsteroidal farnesoid X receptor agonist cilofexor (GS-9674) improves markers of cholestasis and liver injury in patients with primary sclerosing cholangitis. Hepatol. Baltim. Md 70 (3), 788–801. 10.1002/hep.30509 30661255 PMC6767458

[B226] Trivella J. John B. V. Levy C. (2023). Primary biliary cholangitis: epidemiology, prognosis, and treatment. Hepatol. Commun. 7 (6), e0179. 10.1097/HC9.0000000000000179 37267215 PMC10241503

[B227] Turnpenny P. D. Ellard S. (2012). Alagille syndrome: pathogenesis, diagnosis and management. Eur. J. Hum. Genet. 20 (3), 251–257. 10.1038/ejhg.2011.181 21934706 PMC3283172

[B228] Ursic-Bedoya J. Desandré G. Chavey C. Marie P. Polizzi A. Rivière B. (2024). FGF19 and its analog aldafermin cooperate with MYC to induce aggressive hepatocarcinogenesis. EMBO Molecular Medicine 16 (2), 238–250. 10.1038/s44321-023-00021-x 38228803 PMC10897482

[B229] van der Woerd W. L. Houwen R. H. van de Graaf S. F. (2017). Current and future therapies for inherited cholestatic liver diseases. World J. Gastroenterology 23 (5), 763–775. 10.3748/wjg.v23.i5.763 28223721 PMC5296193

[B230] van Munster K. N. Bergquist A. Ponsioen C. Y. (2024). Inflammatory bowel disease and primary sclerosing cholangitis: one disease or two? J. Hepatology 80 (1), 155–168. 10.1016/j.jhep.2023.09.031 37940453

[B231] Vandriel S. M. Li L. T. She H. Wang J. S. Gilbert M. A. Jankowska I. (2023). Natural history of liver disease in a large international cohort of children with alagille syndrome: results from the GALA study. Hepatol. Baltim. Md 77 (2), 512–529. 10.1002/hep.32761 36036223 PMC9869940

[B232] Viana R. J. S. Ramalho R. M. Nunes A. F. Steer C. J. Rodrigues C. M. P. (2010). Modulation of Amyloid-β peptide-induced toxicity through inhibition of JNK nuclear localization and Caspase-2 activation. J. Alzheimer’s Dis. 22 (2), 557–568. 10.3233/JAD-2010-100909 20847398

[B233] Vinayagamoorthy V. Srivastava A. Sarma M. S. (2021). Newer variants of progressive familial intrahepatic cholestasis. World J. Hepatology 13 (12), 2024–2038. 10.4254/wjh.v13.i12.2024 35070006 PMC8727216

[B234] Walker K. F. Chappell L. C. Hague W. M. Middleton P. Thornton J. G. (2020). Pharmacological interventions for treating intrahepatic cholestasis of pregnancy. Cochrane Database Syst. Rev. 2020 (7), CD000493. 10.1002/14651858.CD000493.pub3 32716060 PMC7389072

[B235] Wang S. Xu C. Liu H. Wei W. Zhou X. Qian H. (2023). Connecting the gut microbiota and neurodegenerative diseases: the role of bile acids. Mol. Neurobiol. 60 (8), 4618–4640. 10.1007/s12035-023-03340-9 37121952

[B236] Wang H. Guo Y. Han W. Liang M. Xiao X. Jiang X. (2024). Tauroursodeoxycholic acid improves nonalcoholic fatty liver disease by regulating gut microbiota and bile acid metabolism. J. Agric. Food Chem. 72 (36), 20194–20210. 10.1021/acs.jafc.4c04630 39193771

[B237] Ward J. B. J. Lajczak N. K. Kelly O. B. O'Dwyer A. M. Giddam A. K. Ní Gabhann J. (2017). Ursodeoxycholic acid and lithocholic acid exert anti-inflammatory actions in the Colon. Am. J. Physiology. Gastrointest. Liver Physiology 312 (6), G550–G558. 10.1152/ajpgi.00256.2016 28360029

[B238] Wei S. Ma X. Zhao Y. (2020). Mechanism of hydrophobic bile acid-induced hepatocyte injury and drug discovery. Front. Pharmacol. 11, 1084. 10.3389/fphar.2020.01084 32765278 PMC7378542

[B239] Wilkhu J. S. McNeil S. E. Anderson D. E. Perrie Y. (2013). Characterization and optimization of bilosomes for oral vaccine delivery. J. Drug Target. 21 (3), 291–299. 10.3109/1061186X.2012.747528 30952177

[B240] Willot S. Uhlen S. Michaud L. Briand G. Bonnevalle M. Sfeir R. (2008). Effect of ursodeoxycholic acid on liver function in children after successful surgery for biliary atresia. Pediatrics 122 (6), e1236–e1241. 10.1542/peds.2008-0986 19029197

[B241] Wilson D. M. Cookson M. R. Van Den Bosch L. Zetterberg H. Holtzman D. M. Dewachter I. (2023). Hallmarks of neurodegenerative diseases. Cell 186 (4), 693–714. 10.1016/j.cell.2022.12.032 36803602

[B242] Wolters F. Tolenaars D. van Weeghel M. de Waart D. van de Graaf S. F. J. Paulusma C. C. (2025). The role of bile salts in itch receptor activation. Biochimica Biophysica Acta. Mol. Basis Dis. 1871 (8), 167972. 10.1016/j.bbadis.2025.167972 40628364

[B243] Wu M. Cheng Y. Zhang R. Han W. Jiang H. Bi C. (2024a). Molecular mechanism and therapeutic strategy of bile acids in Alzheimer’s disease from the emerging perspective of the microbiota–gut–brain axis. Biomed. and Pharmacother. 178, 117228. 10.1016/j.biopha.2024.117228 39088965

[B244] Wu Q. Wang Y. Liu J. Guan X. Chang X. Liu Z. (2024b). Microtubules and cardiovascular diseases: insights into pathology and therapeutic strategies. Int. J. Biochem. and Cell Biol. 175, 106650. 10.1016/j.biocel.2024.106650 39237031

[B245] Xing C. Huang X. Wang D. Yu D. Hou S. Cui H. (2023). Roles of bile acids signaling in neuromodulation under physiological and pathological conditions. Cell and Biosci. 13 (1), 106. 10.1186/s13578-023-01053-z 37308953 PMC10258966

[B246] Xu Y. Wang J. Wu X. Jing H. Zhang S. Hu Z. (2023). Gut microbiota alteration after cholecystectomy contributes to post-cholecystectomy diarrhea *via* bile acids stimulating colonic serotonin. Gut Microbes 15 (1), 2168101. 10.1080/19490976.2023.2168101 36732497 PMC9897804

[B247] Yakhine-Diop S. M. S. Morales-García J. A. Niso-Santano M. González-Polo R. A. Uribe-Carretero E. Martinez-Chacon G. (2020). Metabolic alterations in plasma from patients with familial and idiopathic Parkinson’s disease. Aging (Albany NY) 12 (17), 16690–16708. 10.18632/aging.103992 32903216 PMC7521510

[B248] Yang J. I. Yoon J. H. Myung S. J. Gwak G. Y. Kim W. Chung G. E. (2007). Bile acid-induced TGR5-dependent c-Jun-N terminal kinase activation leads to enhanced caspase 8 activation in hepatocytes. Biochem. Biophysical Res. Commun. 361 (1), 156–161. 10.1016/j.bbrc.2007.07.001 17659258

[B249] Yang J. Zhao T. Fan J. Zou H. Lan G. Guo F. (2024). “Structure-guided discovery of bile acid derivatives for treating liver diseases without causing itch,” Cell, 187(25) 7164–7182e18. 10.1016/j.cell.2024.10.001 39476841

[B250] Yeo X. Y. Tan L. Y. Chae W. R. Lee D. Y. Lee Y. A. Wuestefeld T. (2023). Liver’s influence on the brain through the action of bile acids. Front. Neurosci. 17, 1123967. 10.3389/fnins.2023.1123967 36816113 PMC9932919

[B251] Younossi Z. M. Ratziu V. Loomba R. Rinella M. Anstee Q. M. Goodman Z. (2019). Obeticholic acid for the treatment of non-alcoholic steatohepatitis: interim analysis from a multicentre, randomised, placebo-controlled phase 3 trial. Lancet London, Engl. 394 (10215), 2184–2196. 10.1016/S0140-6736(19)33041-7 31813633

[B252] Yu H. Zhao T. Liu S. Wu Q. Johnson O. Wu Z. (2019). MRGPRX4 is a bile acid receptor for human cholestatic itch. eLife 8, e48431. 10.7554/eLife.48431 31500698 PMC6773440

[B253] Zaccai T. C. F. Hassin-Baer S. Kfir N. C. Duell P. B. Neerhof M. Sloma R. (2024). Chenodeoxycholic acid (CDCA) treatment during pregnancy in women with cerebrotendinous xanthomatosis (CTX): lessons learned from 19 pregnancies. Genet. Med. Official J. Am. Coll. Med. Genet. 26 (5), 101086. 10.1016/j.gim.2024.101086 38288684

[B254] Zangerolamo L. Vettorazzi J. F. Rosa L. R. O. Carneiro E. M. Barbosa H. C. L. (2021). The bile acid TUDCA and neurodegenerative disorders: an overview. Life Sci. 272, 119252. 10.1016/j.lfs.2021.119252 33636170

[B255] Zangerolamo L. Carvalho M. Barbosa H. C. L. (2025). The critical role of the bile acid receptor TGR5 in energy homeostasis: insights into physiology and therapeutic potential. Int. J. Mol. Sci. 26 (14), 6547. 10.3390/ijms26146547 40724796 PMC12294878

[B256] Zarrinpar A. Loomba R. (2012). Review article: the emerging interplay among the gastrointestinal tract, bile acids and incretins in the pathogenesis of diabetes and non-alcoholic fatty liver disease. Alimentary Pharmacol. and Ther. 36 (10), 909–921. 10.1111/apt.12084 23057494 PMC3535499

[B257] Zhang M. Zheng J. Nussinov R. Ma B. (2017). Release of cytochrome C from bax pores at the mitochondrial membrane. Sci. Rep. 7 (1), 2635. 10.1038/s41598-017-02825-7 28572603 PMC5453941

[B258] Zhang Y. LaCerte C. Kansra S. Jackson P. J. Brouwer K. R. Edwards J. E. (2017). Comparative potency of obeticholic acid and natural bile acids on FXR in hepatic and intestinal *in vitro* cell models. Pharmacol. Res. and Perspect. 5 (6), e00368. 10.1002/prp2.368 29226620 PMC5723701

